# Estradiol-mediated protection against high-fat diet induced anxiety and obesity is associated with changes in the gut microbiota in female mice

**DOI:** 10.1038/s41598-023-31783-6

**Published:** 2023-03-23

**Authors:** Kalpana D. Acharya, Madeline Graham, Harshini Raman, Abigail E. R. Parakoyi, Alexis Corcoran, Merzu Belete, Bharath Ramaswamy, Shashikant Koul, Ishneet Sachar, Kevin Derendorf, Jeremy B. Wilmer, Srikanth Gottipati, Marc J. Tetel

**Affiliations:** 1 Neuroscience Department, Wellesley College, Wellesley, MA 02481 USA; 2 Otsuka Pharmaceutical Development & Commercialization, Inc., Princeton, NJ 08540 USA; 3 Cornell University, Ithaca, NY 14850 USA; 4 Department of Psychology, Wellesley College, Wellesley, MA 02481 USA

**Keywords:** Neuroscience, Stress and resilience

## Abstract

Decreased estrogens during menopause are associated with increased risk of anxiety, depression, type 2 diabetes and obesity. Similarly, depleting estrogens in rodents by ovariectomy, combined with a high-fat diet (HFD), increases anxiety and adiposity. How estrogens and diet interact to affect anxiety and metabolism is poorly understood. Mounting evidence indicates that gut microbiota influence anxiety and metabolism. Here, we investigated the effects of estradiol (E) and HFD on anxiety, metabolism, and their correlation with changes in gut microbiota in female mice. Adult C57BL/6J mice were ovariectomized, implanted with E or vehicle-containing capsules and fed a standard diet or HFD. Anxiety-like behavior was assessed and neuronal activation was measured by c-fos immunoreactivity throughout the brain using iDISCO. HFD increased anxiety-like behavior, while E reduced this HFD-dependent anxiogenic effect. Interestingly, E decreased neuronal activation in brain regions involved in anxiety and metabolism. E treatment also altered gut microbes, a subset of which were associated with anxiety-like behavior. These findings provide insight into gut microbiota-based therapies for anxiety and metabolic disorders associated with declining estrogens in menopausal women.

## Introduction

Estrogens have profound effects on energy homeostasis in humans and rodents by acting as an anorectic, preventing fat weight gain, and increasing physical activity^1–5^. Lower levels of circulating estrogens in postmenopausal women increase their risk for obesity, type 2 diabetes, cardiovascular disease, and stroke^2,6–9^. In rodents, ovariectomy decreases physical activity and increases food intake^3,10^, while 17β-estradiol (E) treatment in ovariectomized mice fed a HFD prevents weight gain^11–14^, suggesting that E protects against HFD-induced obesity.

In addition to their effects on energy homeostasis, estrogens also exert effects on mood and anxiety in women^15,16^. Postmenopausal women experience an increased rate of depressive and anxious mood^17–20^, particularly during the onset of menopause, that is ameliorated by hormone replacement therapy^21–23^. Similarly, ovariectomy in rodents increases depressive- and anxiety-like behavior^24–27^, while physiological doses of E decrease anxiety-like behavior in ovariectomized rodents, indicating that estrogens have anxiolytic effects in females^28,29^. Estrogen receptor (ER)-specific effects have been demonstrated using ER$\alpha$ and ER $\beta$ knockout mice or receptor-specific agonists, which have shown ER$\beta$ as a primary mediator of anxiety behavior in females^30–32^.

The lower gastrointestinal (GI) tract is inhabited by a collection of bacteria, viruses, archaea, protozoa, and fungi, known as the gut microbiota^33^. These microbes, along with their genomes, comprise the gut microbiome^33^. Mounting evidence indicates that the gut microbiome is integral in maintaining healthy physiology in humans and rodents^34–36^. Dysbiosis of these microbiota has been implicated in metabolic diseases, including obesity^37^ and type 2 diabetes^38,39^. Gut microbiota also primes the development of the innate immune system and host immune response to pathogenic bacteria, and in turn modulate the production of cytokines and lymphokines^40^. Gut immune alterations mediated by bacteria and their metabolites exert effects on the central nervous system via the gut-brain axis^41,42^. Dysbiosis of the gut microbiota is associated with anxiety^43–45^ and depression^46–48^ in laboratory animals and humans. In male rodents, differences in stress sensitivity across strains have been linked to distinct changes in gut microbiota-dependent stress-induced changes in lipid and energy metabolism^49,50^, although these effects have not been explored in females. However, in a different study, female mice challenged with a HFD and chronic mild stress showed less anxiety and a different gut microbial composition compared to males^51^. Collectively, these findings suggest that the gut microbiome profoundly impacts anxiety and metabolism.

Estrogens and diet are independently associated with changes in gut microbiota^52–55^. In rats and mice, ovariectomy shifts the relative abundances of the major phyla, increasing the ratio of Firmicutes to Bacteroidetes. A high relative abundance of Firmicutes relative to Bacteroidetes is associated with metabolic disorders^56–58^. HFD and Western diet (high in fat and sucrose) feeding also alter gut microbiota composition, including changes in relative abundances of Bacteroidetes and Firmicutes in both humans and rodents^59–62^. While HFD-induced alterations in the gut microbiota are associated with changes in anxiety-like and depression-like behavior in male mice^38,63^, it is unknown if E- and diet-induced changes in gut microbiota similarly alter these behaviors in adult female mice.

The aim of this study was to investigate the effects of E and HFD on anxiety-like behavior and energy metabolism and associations with changes in the gut microbiota in female mice. We tested the hypotheses that: (1) E treatment reduces HFD-induced anxiety-like behavior, (2) E alters activity in anxiety-relevant brain regions following stress in female mice on a HFD, and (3) E and HFD-mediated changes in anxiety are associated with alterations in gut microbiota composition.

## Results

### Estradiol prevents HFD-induced obesity and reduces food intake

**Figure 1:**
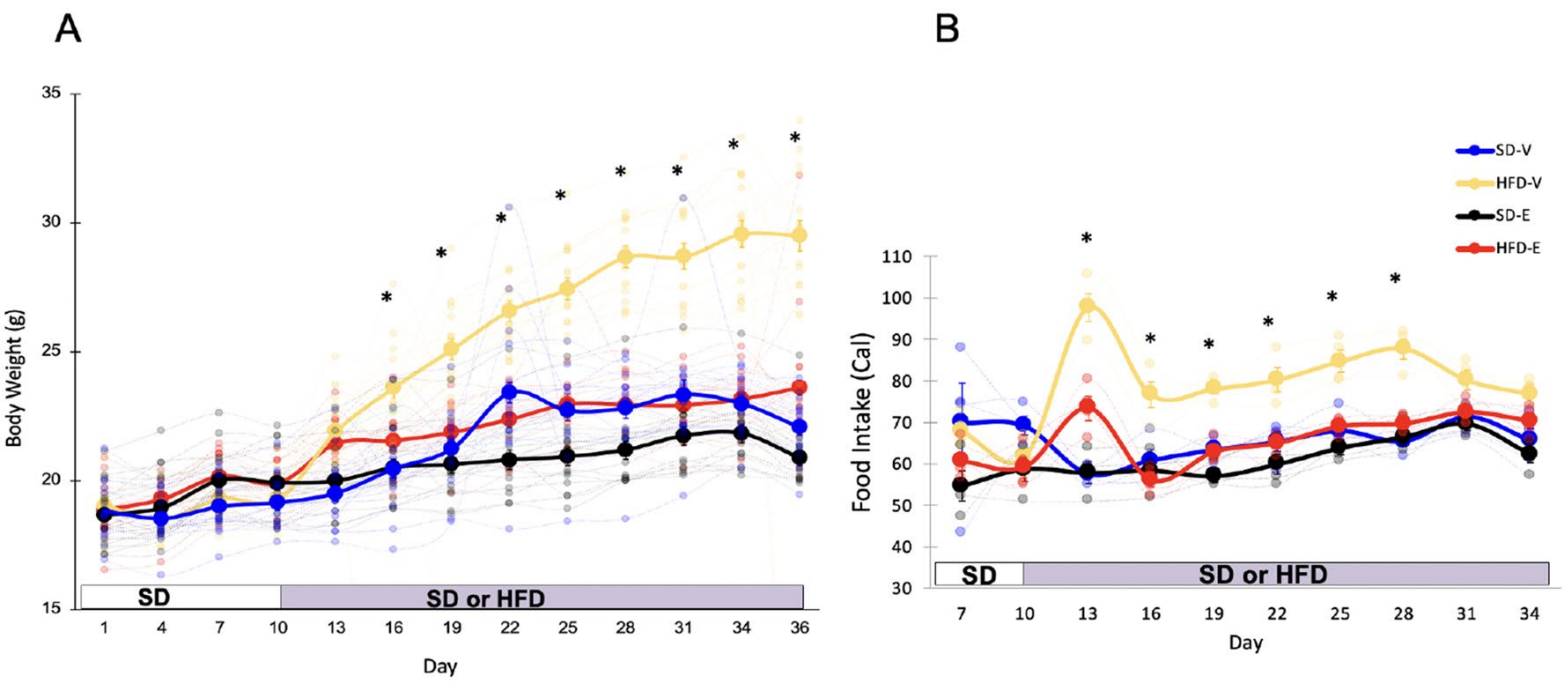
Estradiol prevents HFD-induced obesity and reduces HFD intake in female mice. (**A**) E prevented average body weight gain in mice on a HFD (n = 16/group). (**B**) E decreased caloric intake per cage in mice on a HFD (n = 4 cages/group). Larger circles show mean (± SEM) and smaller circles show individual data points. *Indicate days when HFD-V mice differ from all other groups. *P* < 0.05, Tukey’s HSD.

Eight-week-old C57BL/6J female mice, fed a standard diet (SD) or HFD, were implanted with either 50 μg of 17β-estradiol (E) or oil (V) (Supplementary Fig. 1). Body weight and food intake were measured every three days. Both E and V mice were cohoused with either similar partners (E–E, E-treated mice cohoused with E-treated mice, or V–V) or with mice from a different treatment group (E–V, V–E). Because there were no effects of cohousing on anxiety-like behavior or body weight, cohoused groups were collapsed and effects of diet (SD vs. HFD) and hormone treatment (V vs. E) were analyzed. For the days prior to the diet switch, a one-way repeated measures (RM) ANOVA showed an effect of E (*F*_1,62_ = 4.88, *p* = 0.03) on body weight gain. Following the diet switch, using a two-way RM ANOVA, we found main effects of hormone treatment (*F*_1,57_ = 61, *p* < 0.001), diet (*F*_1,57_ = 89.6, *p* < 0.001) and their interaction (*F*_1,57_ = 24, *p* < 0.001) over time during HFD. During SD feeding, E mice weighed more, starting on D4 (p = 0.018) and continued to weigh more on D7 (p < 0.001) and D10 (p = 0.009), likely due to a faster recovery mediated by E following surgery. On D13, within each hormone condition, HFD groups differed from their SD counterparts. The HFD-V animals weighed more than all other groups starting from day 16 (Tukey’s HSD, *p* < 0.001) (Fig. 1A). The SD-V animals weighed more than the SD-E animals on days 22, 25, and 28 (Tukey’s HSD, *p* < 0.05). The HFD-E mice weighed more than the SD-E animals on days 22, 25, 28, and 36 (Tukey’s HSD, *p* < 0.05).

During the 10 days on SD, one-way RM ANOVA showed an effect of E (*F*_1,14_ = 5.34, *p* = 0.037) on food intake. In particular, E mice ate less than V mice on D10. After switching to HFD, a two-way RM ANOVA found main effects on food intake for diet (*F*_1,12_ = 83.9, *p* < 0.001), hormone treatment (*F*_1,12_ = 51.3, *p* < 0.001), and an interaction between the two (*F*_1,12_ = 24.2, *p* < 0.001). Following the switch to HFD on D10, HFD-V animals consumed the most calories (Tukey’s HSD, *p* < 0.05) except on D31, when food intake for HFD-V showed a strong trend towards an increase compared to HFD-E (p = 0.052) and D34 (Fig. 1B). HFD-E animals ate a similar amount to SD groups throughout the study, except on D13, when they ate more than the SD groups (Tukey’s HSD, *p* < 0.05). Overall, V mice on a HFD weighed more and consumed more calories than all other groups.

### Estradiol reduces anxiety-like behavior

In order to assess the effects of E treatment, diet, and their associations with gut microbiota on anxiety-like behavior, mice were subjected to Light–Dark (LD), Elevated Plus Maze (EPM) and Open Field (OF) testing starting on day 30. The OF test was also administered as a control to confirm that any effects observed in differences in anxiety-like behavior were independent of locomotor activity^64,65^.

### Light–dark test

**Figure 2:**
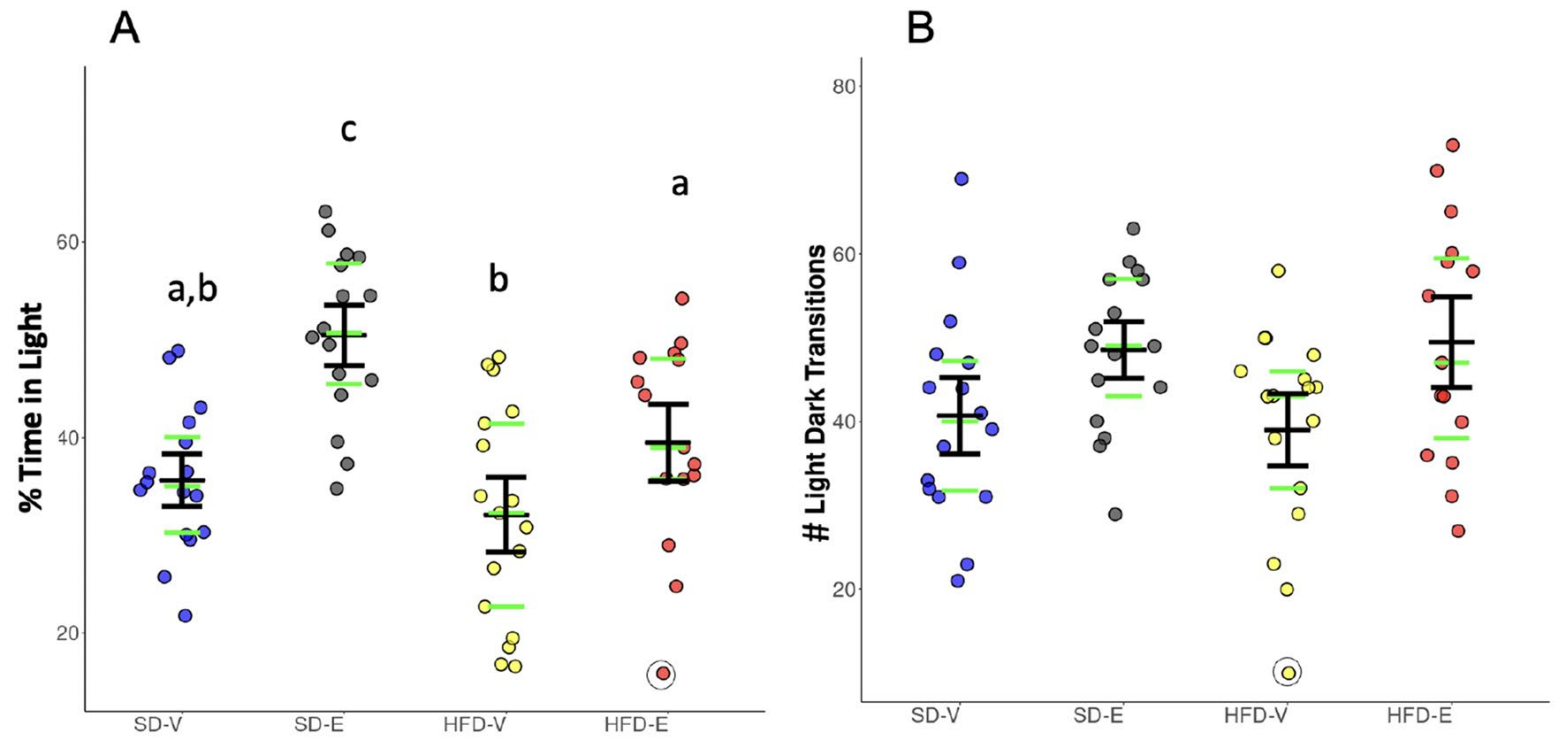
Estradiol reduces anxiety-like behavior in both SD and HFD-fed mice in Light–Dark test (n = 15–16/group). (**A**) Percent (%) time spent in the light compartment. (**B**) The number of light/dark transitions. Black lines show mean (middle line) and 83% CI (whiskers). Green lines show median (middle line) and the 1st (lower line) and the 3rd quartile (upper line). Data points greater than 1.5 times the interquartile range are shown within open circles. Different letters denote differences across groups, *P* < 0.05, Tukey’s HSD.

The LD test was administered to examine anxiety-like behavior based on the anxiogenic effect of light on rodents, as previously described^66,67^. A two-way ANOVA showed main effects for hormone treatment (*F*_1, 60_ = 25.81, *p* < 0.0001) and diet (*F*_1, 60_ = 10.33, *p* = 0.0021) on time spent in the light compartment. E-treated mice spent more time in the light compartment than their vehicle-treated counterparts on SD (Tukey’s HSD, *p* = 0.0002) or HFD (Tukey’s HSD, *p* = 0.045), suggesting that E decreased anxiety-like behavior in both diet conditions (Fig. 2A). HFD-fed E-treated mice spent less time in the light compartment than SD-fed counterparts (Tukey’s HSD, *p* = 0.012), suggesting that HFD had an anxiogenic effect.

There was a main effect of hormone treatment (*F*_1,56_ = 8.68, *p* = 0.0046), but not diet or their interaction, on the number of light–dark transitions. However, Tukey’s HSD post-hoc test found no significant differences between individual groups. These findings suggest the groups did not differ in terms of their tendency to explore the compartments, and thus differences in time spent in the light may be due to differences in aversion to the light (Fig. 2B).

### Elevated plus maze

**Figure 3:**
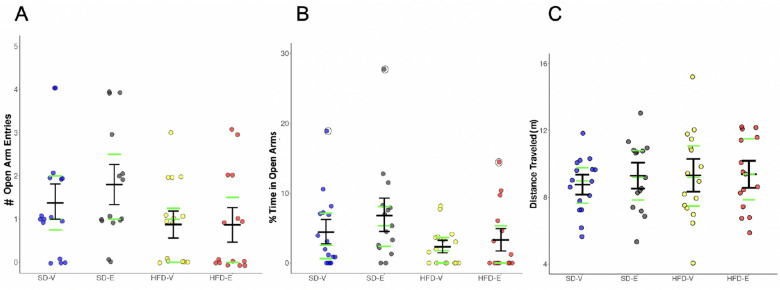
HFD increases anxiety-like behavior in the Elevated Plus Maze test. HFD decreased (**A**) the number of open arm entries, (**B**) time spent in the open arms (n = 15–16/group). For (**A**) and (**B**), a main effect of diet was detected (Kruskal–Wallis, p < 0.05), although individual groups did not differ from each other. (**C**) Total distance traveled. Black lines show mean (middle line) and 83% CI (whiskers). Green lines show median (middle line) and the 1st (lower line) and the 3rd quartile (upper line). Data points greater than 1.5 times the interquartile range are shown within open circles.

The EPM test was used to measure anxiety-like behavior based on rodents’ inherent aversion to open spaces and heights, as previously described^68–70^. While there was an effect of diet (Kruskal–Wallis, p = 0.022) on the number of open arm entries, suggesting that HFD exerted anxiogenic effects in female mice, individual groups did not differ from each other (Fig. 3A). Similarly, there was an effect of diet (Kruskal–Wallis, p = 0.027) on the percent time spent in the open arms, but the individual groups did not differ from each other (Fig. 3B). No effects of diet or hormone treatment were detected on distance traveled on the EPM, suggesting that there were no differences in locomotion that might confound the other measures (Fig. 3C). It should be noted that two animals fell off the maze and were excluded from analysis.

### Open field test

There were no effects of diet or hormone treatment on the number of entries to, or time in the center of the arena, suggesting differences in anxiety-like behavior were not detected by the OF test. Distance traveled was also measured in the OF test to ensure that anxiogenic stimuli were not affecting general locomotor activity as previously described^64,65^. While no main effects of hormone treatment were detected, there was a trend towards an effect of diet (*F*_1,56_ = 3.64, *P* = 0.061) on locomotor activity, as measured by distance traveled in the OF arena. The four treatment groups did not differ in locomotor activity (Supplementary Fig. 2), suggesting there were no differences in general activity across treatment groups. Furthermore, the distance travelled in OF was comparable to previous studies in mice, suggesting the present test parameters did not cause additional stress to the mice^71^.

### Estradiol reduces neural activity in discrete brain regions in HFD-fed animals

**Figure 4:**
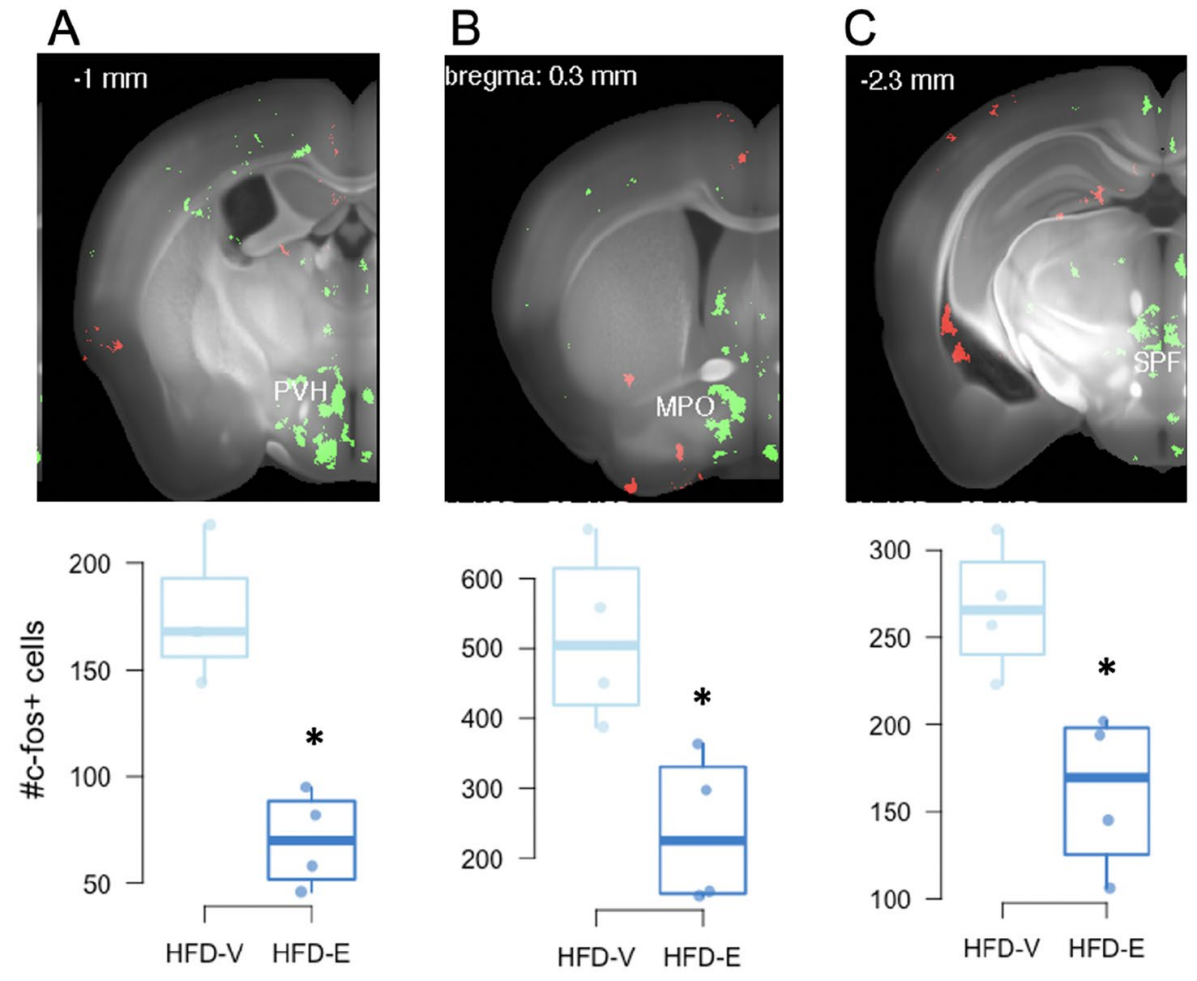
iDISCO reveals that estradiol reduces c-fos immunoreactivity in brain regions involved in anxiety-like behavior and energy homeostasis in HFD-fed female mice. c-fos immunoreactivity (green) in the (**A**) paraventricular nucleus of the hypothalamus (PVH), (**B**) medial preoptic area (MPO), (**C**) subparafascicular nucleus (SPF) of the thalamus (n = 4/group). Some non-specific c-fos immunoreactivity (red) is also observed. The box limits are the first and third quartiles. The whiskers are at 1.5 times the interquartile range below the first quartile and above the third quartile. *Denotes *q* < 0.05 between the two groups.

In order to elucidate the effects of E on neural activity in mice fed a HFD, HFD-E and HFD-V mice were perfused immediately after EPM, the last of the three anxiety tests administered, and brains were immunolabeled for c-fos using iDISCO (Fig. 4). Labeled whole brains were imaged with light sheet microscopy and immunolabeled cells were measured within regions of interest (ROIs) or evenly spaced voxel (each 2 × 2 × 3 µm). E treatment decreased the number of c-fos immunoreactive cells in the paraventricular nucleus (PVH), particularly the medial dorsal parvicellular part (PVHmpd) (*q* < 0.0001), medial preoptic area (MPO) (*q* = 0.03), lateral amygdala nucleus (LA) (*q* = 0.01), and the subparafascicular area (SPA) (*q* = 0.0002), including the subparafasicular nucleus (SPF) (*q* = 0.02) of the thalamus. E also reduced the number of c-fos-immunoreactive cells in the magnocellular nucleus of the anterior bed nucleus of the stria terminalis (BSTmg) (*q* = 0.045).

### Gut microbiota associate with body weight gain

The 16S rRNA from fecal samples was analyzed using Quantitative Insights Into Microbial Ecology (QIIME2) to identify the organisms present in each sample. The taxonomy of each OTU was established by matching to the GreenGenes (v13_8, 97% clustered OTUs) (https://greengenes.secondgenome.com/). Feature tables were used to calculate alpha diversity and a phylogenetic tree was constructed to measure beta diversity metrics.

**Figure 5:**
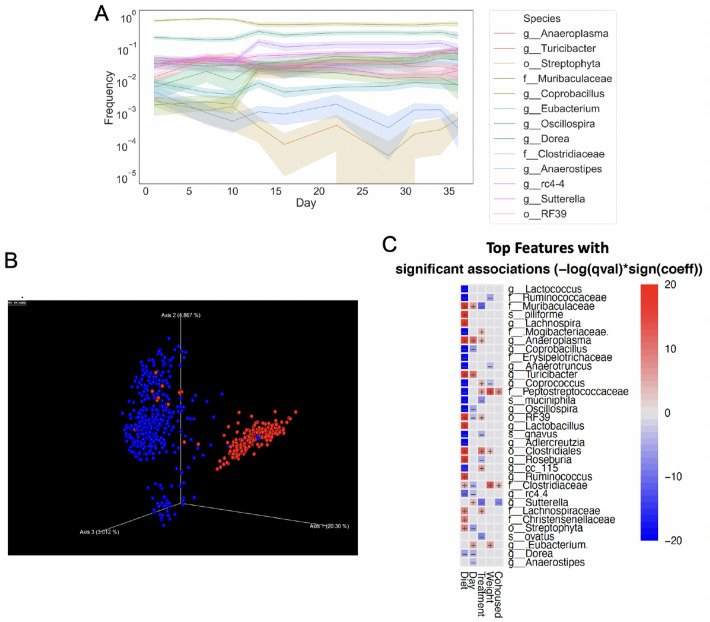
HFD and estradiol alter gut microbial composition. (**A**) Time-longitudinal graph of gut microbiota taxa over time. (**B**) Bray–Curtis dissimilarity show that gut microbiota from SD-fed mice (blue) cluster differently from HFD-fed mice (red) and (**C**) multiple bacterial taxa correlate with E treatment, diet, and body weight in female mice based on longitudinal samples through the full length of the study (n = 16/group). Positive correlation with SD, increased days, V treatment, increased body weight, or cohousing with V mice are shown as red in the heatmap.

Longitudinal composition of gut microbiota based on samples taken from days 16–28 correlated with body weight (Fig. 5). The relative abundances of Clostridiales (order) and its families Peptostreptococcaceae and Clostridiaceae, and *Eubacterium* were positively correlated with body weight gain, of which Peptostreptococcaceae was also positively associated with HFD feeding. Relative abundances of Ruminococcaceae, *Anaerotruncus*, and *Coprococcus* were negatively associated with weight gain, but positively associated with HFD feeding (Fig. 5C).

### Estradiol treatment and HFD feeding alter gut microbiota

16 s rRNA sequence analysis revealed differences in fecal microbiota composition between SD and HFD-fed mice. Longitudinal analysis of gut microbiota revealed a noticeable change in taxa abundance over time, with the largest shift detected on the 5th day on HFD, (D16, Fig. 5A). In particular, there was a sharp decrease in the relative abundances of Muribaculaceae and Streptophyta, whereas *Coprobacillus* and *Oscillospira* increased in response to HFD (Fig. 5A,C). Diet-dependent effect on gut microbiota was additionally confirmed by cluster analysis, using Bray–Curtis distance measure (PERMANOVA, Fig. 5B). Gut microbiota community also clustered differently between E and V groups (*p* < 0.02, Supplementary Fig. 3).

Relative abundances of multiple taxa correlated with E treatment (Fig. 5C). Multivariable correlational analysis found that E treatment was positively associated with Muribaculaceae, *Sutterella, Roseburia,* B. *ovatus, A. muciniphila,* and *R. gnavus.* In contrast, Peptostreptococcaceae, Mogibacteriaceae, *Coprococcus*, and *cc_115* were negatively correlated with E treatment, but positively correlated with HFD feeding. Additional taxa including Clostridiales, Lachnospiraceae, *Anaeroplasma*, and *RF39* were also negatively correlated with E treatment.

HFD-feeding was associated with profound changes in gut microbiota. In addition to the taxa mentioned above that negatively associated with E but positively associated with HFD, Ruminococcaceae, Erysipelotrichaceae, *Coprobacillus, Lactococcus, Anaerotruncus, A. muciniphila, Oscillospira, Adlercreutzia, Dorea, rc 4.4,* and *R. gnavus* were also increased during HFD feeding. In contrast, Clostridiales, Streptophyta, the families Clostridiaceae, Christensenellaceae*,* Muribaculaceae, Lachnospiraceae*, Turicibacter*, *Lactobacillus*, *Lachnospira*, *Ruminococcus*, *Anaeroplasma, Roseburia*, *RF39*, and *C. piliforme* were decreased during HFD intake. Of the microbes that were increased by HFD feeding, Clostridiales and Peptostreptococcaceae were positively associated, while Ruminococcaceae, *Coprococcus,* and *Anaerotruncus* were negatively associated with weight gain.

Cohousing was also associated with alterations in gut microbiota, although, as expected, the effect was not as pronounced when compared with the effect of HFD or E. *Sutterella* was positively associated with cohousing with E mice, while Peptostreptococcaceae and Clostridiaceae were negatively associated (Fig. 5C).

### Gut microbiota associate with anxiety-like behavior

**Figure 6:**
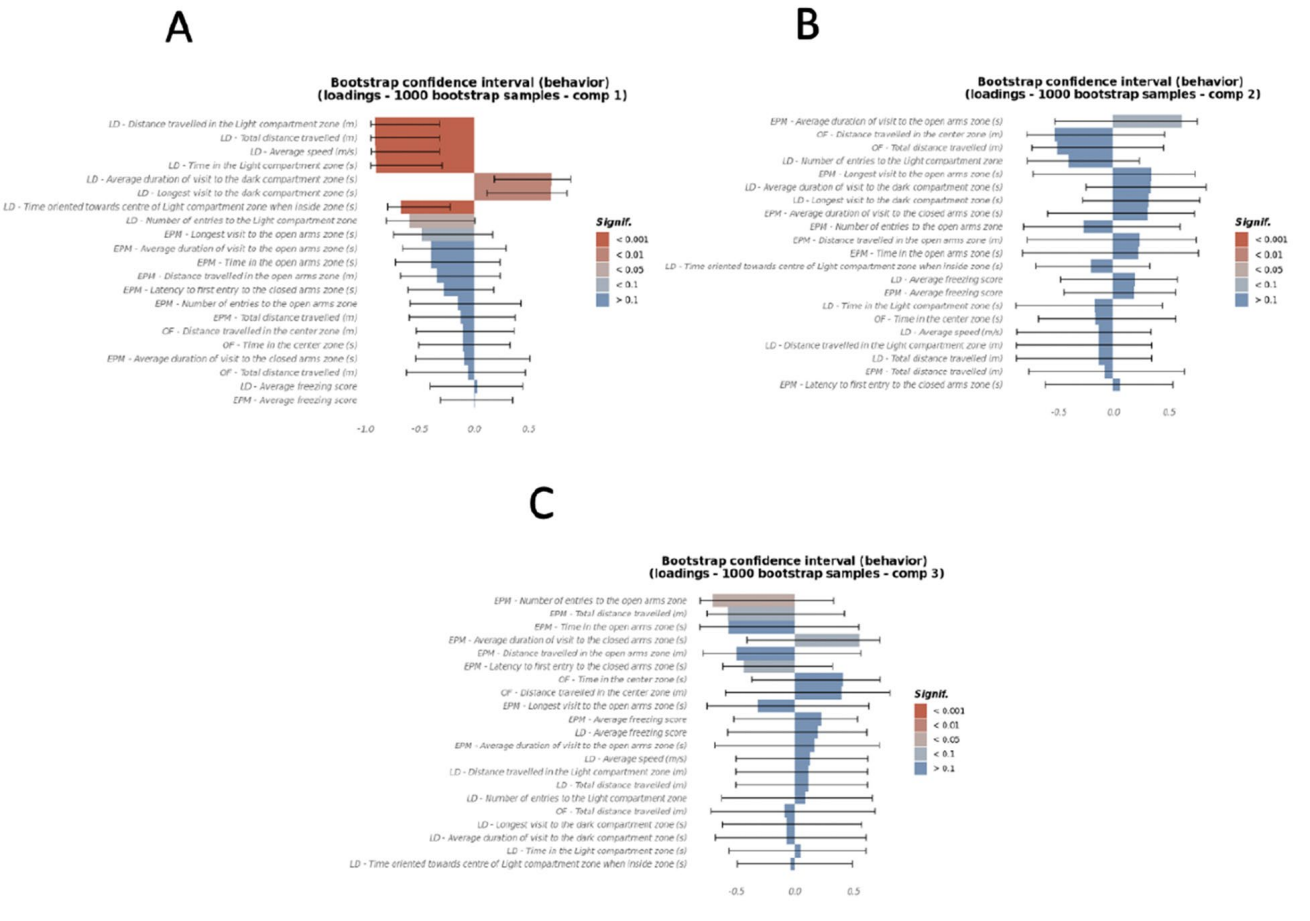
HFD most strongly contributes to anxiety-like behavior in female mice. Canonical loadings of behavioral tests Open Field, Light–Dark, and Elevated Plus Maze tests, were computed using the RGCCA method, of which (**A**) the 1st canonical component that optimized correlation between anxiety behavior and HFD component of the treatment block. (**B**,**C**) The 2nd and 3rd canonical components were orthogonal canonical correlations of anxiety behavior and microbiome with E treatment and cohousing, respectively (n = 8/group). Color gradings depict the statistical significance levels of canonical loadings and whiskers show 95% CI (mean + SEM) which measure the significance and stability of the block-weight vectors on 1000 bootstrap samples. The direction (+/−) of canonical loadings depict the direction (+/−) of variable (anxiety, microbiome, treatment) correlations with canonical variates (anxiety, microbiome, treatment).

Multiblock generalized canonical correlations were computed between measures of the LD, OF, and EPM tests indicative of anxiety-like behavior, longitudinal microbiome features which include abundances on D28, change in abundances between D28 and D13, and change in abundances between D10 and D7, and the treatment binary variables E, diet and cohousing, using the Regularized and Sparse Generalized Canonical Correlation Analysis (RGCCA)^72^. The top 3 canonical components between the three blocks were explored for significant associations (Supplementary Fig. 4A). The first canonical component of the treatment block, which was strongly defined by HFD (adj p = 0.029, CI [0.32, 0.99], Supplementary Fig. 4B), was associated with anxiety (Fig. 6A and Supplementary Table 1) and explained 28% and 12.3% variance on anxiety-like behavior and gut microbiota, respectively (Supplementary Fig. 5A,B). Canonical components 2 and 3 were influenced by E treatment (adj p = 0.11, CI [− 0.22, 0.98]) and cohousing (adj p = 0.023, CI [− 0.99, − 0.01]) respectively (Supplementary Fig. 4C,D and Supplementary Fig. 5C) and each explained 9% and 12% of variance in behavior, respectively (Fig. 6B,C, and Supplementary Figs. 1A and 4A). The correlation of diet and microbiome with increased anxiety behavior was strongly captured by canonical component 1 (Fig. 6A), but to a lesser extent by canonical components 2 and 3 (Fig. 6B,C).

**Figure 7:**
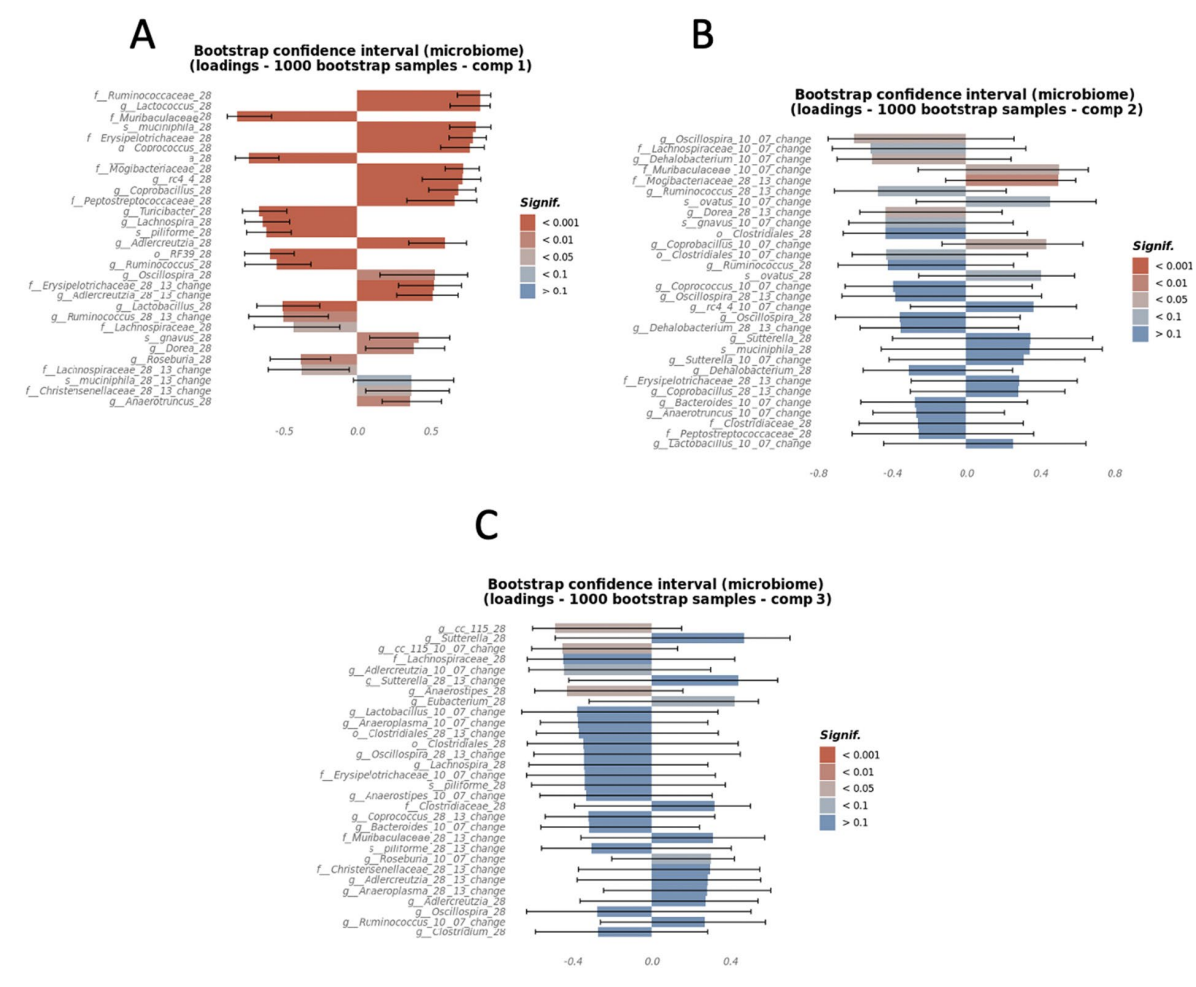
HFD, estradiol and cohouse-induced changes in gut microbiota correlate with anxiety behavior. Canonical loadings of bacterial OTUs of the top 3 canonical components that were associated with diet, estradiol treatment, and/or cohousing, respectively, and were correlated with components of anxiety behavior derived using individual anxiety measures from the Open Field, Light–Dark, and Elevated Plus Maze tests. (**A**) Canonical loadings of microbial taxa that correlated with the 1st canonical component. (**B**,**C**) Canonical loadings of microbes that were correlated with the 2nd and 3rd canonical components of anxiety (n = 8/group). Color gradings depict the statistical significance levels of canonical loadings and whiskers show 95% CI (mean + SEM) which measure the significance and stability of the block-weight vectors on 1000 bootstrap samples. The direction (+/−) of canonical loadings depict the direction (+/−) of variable (anxiety, microbiome, treatment) correlations with canonical variates (anxiety, microbiome, treatment).

To examine the association between changes in bacterial community over time and manifestation of anxiety, changes in microbial abundance during SD (D10 minus D7), and during HFD (D28 minus D13), were correlated with composite anxiety components. Relative abundance of microbial taxa on D28 only (just before anxiety-like behavior testing) were also examined. Increased relative abundances of Muribaculaceae, *Turicibacter*, *Lachnospira*, *C. piliforme*, *RF39*, *Ruminococcus, Lactobacillus,* and *Anaeroplasma* (*p* < 0.0001) and *Roseburia* (*p* < 0.01) on D28 correlated with decreased anxiety-like behavior, as evidenced by their inverse correlation with the canonical component 1 (Fig. 7A). All these microbes were also increased during SD, suggesting that a depletion of SD-associated gut microbial community could increase anxiety.

A net increase in Lachnospiraceae and *Ruminococcus* during HFD feeding, between D13–D28 (*p* < 0.05), was also associated with reduced anxiety-like behavior (Fig. 7A), suggesting that increases in these two taxa may attenuate the detrimental effect of HFD intake on anxiety response.

Multiple microbial taxa were identified to be associated with increased anxiety-like behavior. For example, Ruminococcaceae, Mogibacteraceae, Peptostreptococcaceae, Erysipelotrichaceae, *Lactococcus, A. muciniphila, Coprococcus, rc4_4, Coprobacillus,* and *Adlercreutzia* (*p* < 0.001), and *Oscillospira, R. gnavus, Dorea,* and *Anaerotruncus* (*p* < 0.01) on D28 were positively associated with the anxiety component that was most correlated with both diet and microbiome (Fig. 7A and Supplementary Table 2). These taxa were also associated with HFD, further suggesting that HFD-associated gut microbiota may contribute to anxiety. Similarly, an increase in abundances of Erysipelotrichaceae, *Adlecreutzia* (*p* < 0.001), Christensenellaceae (*p* < 0.05) on D28 compared to D13 was also associated with increased anxiety-like behavior (Fig. 7A and Supplementary Table 2).

The canonical components 2 and 3, which were mostly affected by E treatment and cohousing with E-mice, explained 9% and 12% of the variance in behavior, respectively (Supplementary Fig. 3C,D and Supplementary Fig. 5A)*.* Most gut microbes associated with component 2 were the microbes affected by E, suggesting that microbial alteration resulting from E treatment may have less impact on anxiety (Fig. 7B). Only change in the abundance of Mogibacteriaceae during HFD (D28 minus D13) was notable for having a positive association with the anxiety component that was most correlated with E treatment (*p* < 0.01). However, the confidence interval was wide, suggesting a small sample size. Similarly, cohousing-induced alterations in gut microbiota may also have a limited effect on anxiety (Fig. 7C). None of the alterations were found to be significant based on *p* < 0.01 and had wide confidence intervals.

The first two canonical loadings of the microbiome (Supplementary Fig. 5B) showed a separation based on diet in the first component and E treatment in the second component. The second and third canonical loadings of the microbiome (Supplementary Fig. 5C) showed a separation based on E treatment and cohousing with E-treated mice, respectively. This was also evident in the canonical covariates of the microbiome block, where the first-three components showed a separation based on diet, E treatment and cohousing with E treated mice (Supplementary Fig. 5D–F).

The most significant canonical loadings of the behavior block (Fig. 6A–C) showed a higher representation by LD tests in the first component, mixed representation of LD, EPM and OF in the second component and higher representation of EPM in the third component. Only the first and the third components showed significant correlations with the behavior tests. The first component, that is most associated with the HFD, had a positive association with anxiety behaviors. The third component, negatively correlated with cohousing with E-treated mice, was negatively correlated with number of entries in the open arms zone in EPM. The first canonical covariate of the behavior blocks showed separation based on diet (Supplementary Fig. 5D) but not as wide as the first canonical covariate of the microbiome, possibly due to the difference in correlation between microbiome and behavior blocks with the diet (Supplementary Fig. 4A). Additionally, there was a different trend between the interaction of E treatment and cohousing in the HFD compared to SD in the first canonical covariate of the behavior block. The second and third canonical covariates of the behavior block showed a reverse trend when compared to the second and third canonical covariates of the microbiome block (Supplementary Fig. 5E,F), possibly due to the different direction of correlation between microbiome and behavior blocks with the treatment block (Supplementary Fig. 4A): the microbiome components had a positive correlation, and the behavior components had a negative correlation.

## Discussion

In the present study, we investigated the impact of E and diet on body weight, gut microbiota, and anxiety-like behavior. E treatment reduced HFD intake and prevented HFD-induced obesity in female mice, consistent with our previous findings^11,12,58,73^ and those of others^14,74,75^. Moreover, the present findings reveal that E reduces anxiety-like behavior and neural activity in discrete brain regions associated with energy homeostasis and anxiety processing. Consistent with our previous studies and others^51,58,76^, HFD dramatically alters gut microbiota composition in female mice. E treatment associated with attenuation of some of these HFD-induced changes in gut microbiota. Moreover, HFD-induced changes in gut microbiota were associated with increased anxiety-like behavior. These findings suggest that E functions to maintain energy metabolism and modulate anxiety in female mice challenged with a HFD by attenuating the HFD-induced changes in gut microbiota.

### HFD and estradiol affect anxiety-like behavior

In the present study, E reduced anxiety-like behavior in mice on SD and HFD in the LD test, suggesting E has an anxiolytic effect in both diet conditions. This increase in anxiety-like behavior was not due to altered locomotor activity^64,65^, as locomotor activity was not different between hormone groups in the OF test. However, no differences in anxiety-like behavior were detected in mice on SD or HFD in the EPM or OF tests. In support of E reducing anxiety-like behavior as detected in the LD test, estrogens influence anxiety in humans and rodents by modulating the activity of the hypothalamic–pituitary–adrenal axis and serotonergic system^28^. Both anxiogenic and anxiolytic effects of E have been reported in female rodents, depending on timing, dose, and the nature of the behavioral testing and the anxiogenic stimuli used^25,77,78^. These opposing effects of E administration on anxiety-like behavior are likely due to the diverging action of different estrogen receptor (ER) subtypes, with ERβ and ERα activation having mostly anxiolytic and anxiogenic effects, respectively^31,79^***.*** The present findings revealed that HFD had an anxiogenic effect in female mice, consistent with other studies in female and male mice^80,81^. Furthermore, we found that numerous microbial communities increased by HFD were associated with increased anxiety-like behavior, suggesting a mechanism by which HFD influences anxiety-like behavior. In support, changes in gut microbiota have been implicated as a mediating factor in diet-induced obesity (DIO)-related changes in anxiety-like behavior^38^.

### HFD and estradiol alter neuronal response in brain regions involved in anxiety

Using iDISCO, whole brains from animals on HFD were analyzed for c-fos expression, an indicator of neuronal response^82^, at the end of the experiment following the three anxiety-like behavior tests. As discussed below, E decreased c-fos expression in a number of brain regions involved in anxiety-like behavior and the stress response. While it is not known if this reduction in c-fos expression by E is affecting anxiety-like behavior, these findings do suggest that E altered neuronal responses in these brain regions involved in anxiety following the three behavior tests. E treatment decreased c-fos expression in the PVH, which functions in coordinating many autonomic processes including the stress response, energy homeostasis and reproduction^83–85^. In particular, compared to control mice, E-treated mice had a robust decrease in c-fos expression in the medial parvicellular part of the dorsal zone (PVHmpd), a brain region critical in driving the release of adrenocorticotropic hormone and activating the hypothalamic–pituitary–adrenal axis^85^. In rats, ERβ activation by an isoform-specific ligand decreases neural activity in the PVH in response to stress^86,87^, suggesting that ERβ activation decreased anxiety-like behavior and c-fos expression in the present study. E treatment also decreased c-fos expression in the subparafasicular nucleus (SPF). The SPF projects to areas involved in anxiety, including the amygdala, BST, hypothalamus and medial prefrontal cortex^88^. Further implicating the SPF in anxiety, SPF neurons produce tuberoinfundibular peptide of 39 residues, a peptide involved in anxiety-like behavior in rodents^89,90^. E treatment also decreased c-fos expression in the MPO of mice fed a HFD. ERα activation in the MPO has been implicated in anxiety-like behavior in female rodents, with ERα knockdown decreasing anxiety-like behavior in rats^91^. Taken together, these findings suggest that E-induced reduction of neural activity in the SPF and MPO decreases anxiety-like behavior in female mice. In future studies, it will be important to compare c-fos expression following anxiety-like behavior tests in animals fed standard diet vs. HFD.

### Gut microbiota associate with anxiety-like behavior

In addition to the central effects of estrogens on anxiety discussed above, there is evidence that estrogens and HFD impact the gut microbiota to alter communication between the gut and central nervous system via the gut-brain axis^44,45^. Thus, it is important to consider the possible effects of the changing gut microbiome on anxiety. In the present study, microbes increased by HFD, including Ruminococcaceae, Mogibacteraceae, Peptostreptococcaceae, Erysipelotrichaceae, *Lactococcus, A. muciniphila, Coprococcus, Coprobacillus, Adlercreutzia,* and *Oscillospira,* were associated with increased anxiety-like behavior. Of these, Mogibacteraceae, Peptostreptococcaceae, and *Coprococcus* were decreased in E mice, suggesting that HFD-induced proliferation of these microbial communities increases anxiety risk and is exacerbated by reduction in estrogens. In particular, Erysipelotrichaceae and its genus *Coprobacillus,* which were increased by HFD and E-deficiency have been shown to be associated with weight gain^58,73,92^, suggesting a mechanism by which menopause leads to weight gain and increased anxiety.

*Akkermansia* is a gut microbe that has been associated with metabolic health and as having a beneficial role in the stress response. Male mice exposed to chronic social defeat stress had a lower abundance of *Akkermansia*, suggesting that decreased levels of this microbe are associated with increased anxiety^93,94^. However, interestingly, *Akkermansia* was positively associated with increased anxiety in female mice in the current study. These differing results between previous studies and the present findings could be due to a sex difference^95^. Since *Akkermansia* is increased by HFD feeding, the detrimental effects of HFD feeding on anxiety could be augmented by *Akkermansia* and other microbial communities as a result of HFD intake. Interestingly, *Akkermansia* is increased in a rat model of Type 1 diabetes mellitus in females, providing further support for its association with metabolic and inflammatory perturbation in females^96^. In future studies it will be important to investigate sex differences in the function of *Akkermansia* and other microbes in behavior.

Gut microbes that were dominant in SD-fed mice, including Muribaculaceae, *Turicibacter*, *Lachnospira*, *C. piliforme*, *Ruminococcus, Lactobacillus, Roseburia,* and *Anaeroplasma* were negatively correlated with anxiety-like behavior. *Turicibacter,* which was associated with decreased anxiety, was associated with SD in the current study and a previous one^73^, suggesting it as a promising candidate in low-fat diet-dependent mediation of metabolic health and mood. Moreover, a *Lactobacillus* species, *L. rhamnosus*, decreased anxiety and increased sociability in female pups. The *L. rhamnosus-*dependent decrease in anxiety was accompanied by a decrease in Erysipelotrichaceae^97^. In the present study, Erysipelotrichaceae was increased due to HFD and E-deficiency, and positively associated with anxiety. Quorum sensing, or cross-talk among microbial species, may be the mechanism by which these bacterial population densities are regulated^98^.

### Specific gut microbial taxa associate with body weight gain

In the present study, a total of 33 bacterial taxa associated with weight gain, E treatment, cohousing, and diet. Among these are key taxa including Muribaculaceae, which negatively correlate with HFD feeding and overweight phenotypes^58,73,99,100^. Many of the constituent microbes of Muribaculaceae produce short-chain fatty acids (SCFA) that protect from inflammation and metabolic endotoxemia through bacterial fermentation of dietary fiber, suggesting these microbes promote a healthy metabolic milieu and function in mediating E-induced protection from diet-induced obesity^101,102^. In contrast, an increase in Peptostreptococcaceae in V mice during HFD intake and its association with weight gain is consistent with previous findings in female mice^58,73^, suggesting this microbe contributes the detrimental effects of high fat diet intake.

The relative abundance of *A. muciniphila* positively associated with E treatment and HFD feeding, suggesting it plays a role in mediating estrogenic protection from HFD-induced obesity. In support, *A. muciniphila*, the only species of the family Verrucomicrobiaceae cultured from intestinal contents, is associated with healthy body weight and energy metabolism in humans and rodents^103–105^. In the gut, *A. muciniphila* digests mucin and produces SCFAs, suggesting that it has an important function in maintaining gut epithelial integrity and function^106^. Furthermore, *A. muciniphila* abundance correlated with low adiposity, insulin sensitivity, and improved other markers of energy metabolism in male and ovariectomized female mice treated with E^57^. Administration of either live or heat-killed *A. muciniphila* protected from and rescued DIO and metabolic endotoxemia in male mice, indicating a causal role in preventing weight gain and inflammation^107,108^. Considering the correlation of *Akkermansia* with increased anxiety in the current study, and given that much of the previous work has been done in males, it will be important for future work to explore the effects of *A. muciniphila* administration on metabolic physiology and mood in female mice.

### Estradiol alters neural activity in brain regions involved in metabolism

Work from our lab and others has shown that E protects female mice from HFD-induced obesity^11–14^. Estrogens act centrally in the brain^14,109,110^ and peripherally^13,75,111^ to regulate energy homeostasis and protect against HFD-induced obesity. In the present study, E-mediated protection from HFD-induced obesity was in part due to its anorectic effects as E-treated mice consumed less HFD than vehicle mice. Although their food intake did not differ, the SD-V mice weighed more than the SD-E mice at the end of the study (e.g. days 22, 25, and 28), suggesting that E alters energy homeostasis on a standard diet. In support, ovariectomized mice gain more weight than intact female mice even when food intake is similar or less than intact animals^112,113^.

Estrogenic action in the hypothalamus plays an integral role in regulating many aspects of metabolism, including feeding^3^. In the present study, E-treated HFD-fed mice had a decreased number of c-fos immunoreactive cells in the PVH, and MPO compared to V control mice, suggesting E reduced neural activity in these brain regions. The PVH is critical in regulating many aspects of energy homeostasis, including food intake. Lesions to the PVH lead to hyperphagia and obesity in female rodents^114,115^, while E implants in this region reduce food intake in ovariectomized rodents^116^. Similarly, the MPO is important in energy homeostasis, with infusion of E into the MPO reducing food intake in ovariectomized rats^117,118^. We have previously shown that E reduces food intake and weight gain in ovariectomized mice fed a HFD and that E and HFD alter hypothalamic neurogenesis^11,12^. Taken together, the present findings suggest that E-mediated changes in activity in these key hypothalamic regions protect female rodents from HFD-induced obesity. The present study identified changes in gut microbiota and anxiety-like behavior in E-treated groups. In addition, E replacement persistently attenuated calorie intake during HFD feeding. Thus, the possibility that some of these changes in gut microbiota and anxiety are driven by excess calorie intake should be addressed in future studies using pair-feeding paradigms.

### Summary

The present findings provide evidence that E and gut microbiota mediate HFD-induced obesity and anxiety in female mice. The identification of microbial taxa that correlate with protection from diet-induced obesity and anxiety is critical in understanding the peripheral mechanisms by which E regulates mood and energy homeostasis. Given mounting evidence that psychiatric^119^ and metabolic^39,120^ disorders entail gut microbial dysbiosis, understanding how estrogenic modulation of mood and energy metabolism associates with gut microbial composition and function will enhance our understanding of host physiology. Taken together, these findings provide a basis for further exploration of these gut microbes through functional studies to elucidate their specific roles. Most importantly, these results allow identification of microbial targets for the comprehensive treatment of metabolic and mental health disorders, as well as other endocrinopathies that affect women’s health.

## Methods

### Animals

Eight-week-old C57BL6 female mice (Jackson Laboratories, Bar Harbor, ME) were housed two per cage and maintained under a 12:12 h light/dark cycle (lights on 100 h to 1300 h) and fed a standard chow diet (SD) consisting of 13.5% kcal from fat (catalog #5001; Purina, St. Louis, MO). Mice were anaesthetized with 2.5% isoflurane, bilaterally ovariectomized (OVX) and implanted with a capsule made of Silastic tubing (Dow Corning, Midland, MI) capped with silicone sealant^121^ containing either 50 μg of 17β-estradiol (E) dissolved in 25 μl of 5% ethanol/sesame oil (E mice, n = 32) or vehicle (5% ethanol/sesame oil) (V mice, n = 32)^122,123^. The capsules were implanted subcutaneously just below the left scapular blade. Nine days after surgery, mice were started on a high fat diet (HFD) consisting of 60% kcal from fat in the form of lard and soybean oil (26.2% protein, 25.6% carbohydrate, 34.9% fat by weight) (catalog #D12492, Research Diets Inc., New Brunswick, NJ) or maintained on SD. In order to assess the effects of fecal microbiota exchange via coprophagy, mice were cohoused in three different configurations; E and E, E and V, and V and V.

Body weight and food intake were measured every three days at 900–1100 h. Fresh fecal samples were collected on the same days when mice were weighed and were immediately stored at − 80 °C until DNA extraction. On days 30–36, anxiety-like behavior of mice was assessed by: Light–Dark (LD), Open Field (OF), and Elevated Plus Maze (EPM) tests (Supplementary Fig. 1). All animal procedures were approved by the Institutional Animal Care and Use Committee of Wellesley College and were done in accordance with the NIH Animal Care and Use Guidelines.

### Anxiety-like behavior tests

In order to assess the effects of E, HFD and the resulting changes in gut microbiota on anxiety-like behavior, mice were subjected to a battery of anxiety tests over 7 days at the end of the study. All mice were habituated in the testing room for 2 h prior to testing. All tests were recorded and analyzed by ANY-maze software (version Stoelting, Wood Dale, IL). All testing apparatuses were cleaned with hypochlorous water and allowed to dry between trials to disinfect and reduce odors from the mice tested in the previous trials.

### Light–dark test

The anxiogenic effects of light on rodents were measured using the LD test as previously described^66,67^. The LD box (40 × 40 × 35 cm) is divided into two compartments of equal size separated by a wall of black Perspex with a small opening connecting them (#63101, Stoelting, Wood Dale, IL). The light compartment is made of clear Perspex and was illuminated to 300 lx. The dark compartment is made of black Perspex and was illuminated to < 5 lx. Mice were tested during the dark phase, beginning 2 h after lights off. The 10-min test was initiated by placing a mouse in the center of the light compartment facing the dark compartment. Number of transitions between light–dark compartments, latency to enter the dark compartment, latency to reenter the light, distance traveled in the light compartment, total distance traveled, average speed, time spent in either compartment, and average freezing score were measured.

### Open field test

In order to assess locomotor activity and anxiety-like behavior, mice were subjected to the OF test as previously described^64,65^. The OF apparatus is a beige ABS plastic enclosure divided into four 50 × 50 × 38 cm compartments (Wellesley College Machine Shop, Wellesley, MA). The center zone of the apparatus was defined as the 30 × 30 cm square area in the center of the apparatus. On day 33 post-OVX/implant, mice were tested two at a time in two adjacent compartments, beginning 2 h after lights off. The apparatus was illuminated at 120 lx. The 10 min test was initiated by placing a mouse in the center of the apparatus. The behavior was recorded and total distance traveled, entries to the center zone and time and distance travelled in the center zone were measured.

### Elevated plus maze test

Anxiety-like behavior was assessed using the EPM test based on rodents’ inherent aversion to open spaces and heights as previously described^68–70^. The EPM is a beige ABS plastic plus-shaped maze elevated 50 cm with two open arms and two closed arms (35 × 5 cm) with 15 cm walls that intersect at the open center (Wellesley College Machine Shop). Testing was conducted during the light phase, beginning 2 h after lights-on in normal room lighting (100 lx)^70^. The 5 min test was initiated by placing a mouse in the center of the maze facing an open arm. The behavior was recorded and open arm entries, distance traveled, and percent time spent in the open arms, average duration of and longest visit to the open arms, latency to first enter the closed arms, total distance travelled, and average freezing score were measured.

### c-fos Immunolabeling by iDISCO+

In order to observe the effects of E on neural responses to anxiety in mice on a HFD, brains of HFD-V and HFD-E (*n* = 4/group) mice were labelled for c-fos, a marker for neuronal response^82^ by modified immunolabeling-enabled three-dimensional imaging of solvent-cleared organs (iDISCO+, Certerra, Inc., Farmingdale, NY)^124^. On day 36, mice were deeply anesthetized with an intraperitoneal injection of Fatal-Plus (sodium pentobarbital; 390 mg/ml, 100 μl) and perfused transcardially with 0.01 M phosphate buffered saline (PBS) pH 7.2, for one minute followed by 4% paraformaldehyde (w/v) for 8 min at a flow rate of 8 ml/min, 1.5–3 h after EPM testing. Brains were dissected out, post-fixed in 4% paraformaldehyde overnight, transferred to 0.1 M glycine in phosphate buffer for 48 h, and stored in 0.01 M PBS at 4 °C until processing.

Brains were then dehydrated in a graded series of methanol/water solutions (20%, 40%, 60%, 80%), and washed with 100% methanol twice for 1 h each. Brains were bleached overnight in 5% hydrogen peroxide (H_2_O_2_) in methanol (1 volume of 30% H_2_O_2_ for 5 volumes of methanol, ice cold) at 4 °C. Brains were rehydrated with a graded series of methanol/water solutions (80%, 60%, 40%, 20%), then washed with 0.01 M PBS with 0.2% TritonX-100 twice for 1 h each at room temperature. Tissue was permeabilized with 0.1 M PBS with 0.2% TritonX-100, 20% dimethyl sulfoxide (DMSO), and 0.3 M glycine for 36 h at 37 °C. Brains were blocked in 0.1 M PBS and 0.2% TritonX-100 with 10% DMSO and 6% donkey serum for 2 days at 37 °C. Brains were incubated with rabbit anti-c-fos (9F6) monoclonal antibody (#mAb2250, Cell Signaling Technology, Beverly, MA) at 1:400 in PBS-Tween 0.2% with heparin 10 µg/ml with 5% DMSO and 3% donkey serum at 37 °C for 7 days. The brains were washed with the PBS-Tween 0.2% with heparin 10 µg/ml for 24 h (5 changes) and incubated with donkey anti-rabbit-Alexa 560 at 1:450 in PBS-Tween 0.2% with heparin 10 µg/ml and 3% donkey serum at 37 °C for 7 days. Finally, brains were washed with PBS-Tween 0.2% with heparin 10 µg/ml before being cleared.

Brains were once again dehydrated with a graded series of methanol/water solutions (20%, 40%, 60%, 80%, 100% twice) for 1 h each at room temperature. Brains were then incubated in 66% dichloromethane/33% methanol at room temperature for 3 h and then incubated in 100% dichloromethane to wash away the methanol with shaking for 15 min, twice. To finish clearing, brains were incubated with dibenzyl ether (DBE, #108014, Sigma) for 30 min, and then stored in DBE.

### Imaging and quantification of c-fos positive cells

Cleared and labeled whole brains were imaged in the sagittal orientation with a light sheet fluorescence microscope (LSFM) (Ultramicroscope II, LaVision Biotech, Bielefeld, Germany) with a sCMOS camera (Andor Neo) and a 4x/0.5 objective lens (MVPLAPO 4x) with a 6 mm working distance dipping cap. Brains were imaged in 3 µm optical sections at 2 × 2 × 3 µm voxel resolution. The brain images were aligned and c-fos-positive cells were detected and counted. iDISCO c-fos positive cell counts were statistically compared by negative binomial regression. Statistical comparisons between the two groups were run based on either regions of interest (ROIs) or evenly spaced voxels. Voxels are overlapping 3D spheres with 100 μm diameter each and spaced 20 μm apart from each other. The cell counts at a given location, *Y*, were assumed to follow a negative binomial distribution whose mean is linearly related to one or more experimental conditions, *X:* *E[Y]* = *α* + *βX*. For example, when testing an experimental group versus a control group, the *X* is a single column showing the categorical classification of mouse sample to group id, i.e. 0 for the control group and 1 for the experimental group^125,126^. The maximum likelihood coefficients *α* and *β* through iterative reweighted least squares were determined, obtaining estimates for sample standard deviations in the process, from which the significance of the *β* coefficient was obtained. A significant *β* means the group status is related to the cell count intensity at the specified location. Z-values correspond to this *β* coefficient normalized by its sample standard deviation, which under the null hypothesis of no group effect, has an asymptotic standard normal distribution. The p-values reveal the probability of obtaining a *β* coefficient as extreme as the one observed by chance assuming this null hypothesis is true. To account for multiple comparisons across all voxel/ROI locations, p-values were thresholded at 0.1 and false discovery rates with the Benjamini–Hochberg procedure were reported as adjusted q-values^127^.

### 16s rRNA sequencing and analysis

DNA was extracted from frozen fecal samples collected on days 1, 4, 7, 10, 13, 16, 22, 28, 31, 34, and 36 using the DNeasy PowerSoil Kit (Qiagen, Germantown, MD) with minor adjustments to the provided protocol. The lysis step was performed using a FastPrep-24™ 5G Instrument (MP Biomedicals, Santa Ana, CA). A 5-min incubation with 50 µl of elution buffer C6 before centrifugation was added to increase final concentration. Quality and quantity of DNA were measured using a Nanodrop spectrophotometer (Thermo Scientific, Waltham, MA). Extracts were stored at − 20 °C before amplification and sequencing.

The V3-V4 region of the 16S rRNA gene was amplified in the samples using the forward Nextera Meta_V4_515 (5′-TCGTCGGCAGCGTCAGATGTGTATAAGAGACAGCCTACGGGA GGCAGCAG-3′) and reverse Meta_V4_806 (5′-GTCTCGTGGGCTCGGAGATGTGTATAAGA GACAGGGACTACHVGGGTWTCTAAT-3′) primers with flow cell adapters on each. The indexed PCR products were quantified using PicoGreen (Invitrogen, Carlsbad, CA). Once quantified, the amplicons were normalized to 10 ng/µl, pooled, and purified using SPRI purification. The cleaned amplicons were then quantified by Qubit (Invitrogen, Carlsbad, CA) and underwent fragment analysis by Agilent TapeStation (Agilent, Santa Clara, CA). The pooled samples were sequenced with a paired-end Illumina MiSeq 600 cycle v3 kit (Illumina, San Diego, CA).

Forward and reverse reads were merged for each sample. The demultiplexed raw amplicon sequences were processed using an open-source software package of Quantitative Insights Into Microbial Ecology (QIIME2)^128^. Denoising and dereplication of paired-end sequences including chimera removal and trimming of reads based on positional quality scores were performed using the Divisive Amplicon Denoising Algorithm 2 (DADA2) built in QIIME2, an amplicon-specific error-correction method that models and corrects Illumina-sequenced amplicon errors^129^. Briefly, a feature table containing counts of each unique sequence variant in the samples was constructed using DADA2 as an operational taxonomic unit (OTU). An OTU is a cluster of sequences that differ by less than 3% dissimilarity in our analysis.

In order to calculate alpha diversity metrics including observed feature counts (or observed OTUs) and Shannon and Simpson diversity indices, the feature table containing OTUs was rarefied. We further calculated microbial beta diversity (Bray–Curtis distance) using PERMANOVA. A summary of beta diversity relationships was visualized using principal coordinate analysis as PCoA plots.

Taxonomic composition analysis was performed to identify the organisms present in each sample. The taxonomy of each OTU was established by matching to the GreenGenes (v13_8, 97% clustered OTUs), {https://greengenes.secondgenome.com/, #988} based on a naive Bayesian classifier with default parameters of QIIME2^130–132^.

### Body weight, food intake, and anxiety-like behavior data analyses

Body weight, food intake, behavior measures, and microbiota data were analyzed in Jamovi (v 1.8.4.0) or R (The R Foundation of Statistical Computing, v3.5.1) using the ‘lme’ (from the package ‘nlme’, v 3.1-152) and ‘anova (base R)’ functions with a repeated measures design in both one-way (by groups), two-way (diet and hormone treatment), or three-way (diet, hormone, and cohousing) analyses. For analyzing normally distributed single timepoint body weight, food intake, and behavior measures, a three-way (diet, treatment, and cohouse) or a two-way ANOVA (diet and treatment) were used (Jamovi). When there was a significant difference (*P* < 0.05), Tukey’s honestly significant difference (HSD) post-hoc test was used for comparisons. Some behavioral measures violated assumptions of normality and homogeneity of variance, thus were analyzed using non-parametric Kruskal–Wallis test followed by DCSF pairwise comparisons with family-wise corrections (Jamovi) and confidence intervals were generated using bootstrapping with 10,000 replicates, using ShowMyData.org (v.2.0)^133^.

### Longitudinal microbiota correlational analysis

The optimization problem is defined in Eq. (1) (Supplementary Fig. 6). **X**_***i***_ and **a**_***i***_ denote the block data matrix and the weights, and the subscripts *t*, *b* and *m* denote treatment, behavior and microbiome respectively. Absolute value of covariance represents the centroid scheme. Each block is connected, i.e., the design matrix is an identity function (the coefficients of the covariances in the optimization are equal to 1). *τ*_*i*_ denotes the shrinkage that can be adjusted between maximum correlation (for *τ*_*i*_ = 0) and maximum covariance (for *τ*_*i*_ = 1).

1 $$\begin{array}{c}\underset{{a}_{t},{a}_{b},{a}_{m}}{\mathrm{max}}\left|{\text{cov}}\left({\mathbf{X}}_{t}{\mathbf{a}}_{t},{\mathbf{X}}_{b}{\mathbf{a}}_{b}\right)\right|+\left|{\text{cov}}\left({\mathbf{X}}_{b}{\mathbf{a}}_{b},{\mathbf{X}}_{m}{\mathbf{a}}_{m}\right)\right|+\left|{\text{cov}}\left({\mathbf{X}}_{t}{\mathbf{a}}_{t},{\mathbf{X}}_{m}{\mathbf{a}}_{m}\right)\right|\\ \text{s.t.}\left(1-{\tau }_{i}\right){\text{var}}\left({\mathbf{X}}_{i}{\mathbf{a}}_{i}\right)+{\tau }_{i}\parallel {\mathbf{a}}_{i}{\parallel }^{2}=1\text{ where }i\in \{t,b,m\}\end{array}.$$

The treatment block, **X**_***t***_, comprised of estradiol treatment (E), high-fat diet (HFD) and cohousing (C) binary vectors. Individual components for the LD, OF, and EPM behavioral tests indicative of anxiety-like behavior comprised the behavior block, **X**_***b***_. Microbiome block, **X**_***m***_, comprised of 3 derived features from observed longitudinal microbiome abundances: pre-HFD diet relative abundance as derived by change between day 10 and day 7 taxa abundances, post-HFD diet relative abundance as derived by change between day 28 and day 13 taxa abundances and lastly, relative abundance on day 28.

The centroid scheme was used as the scheme function to enable two components to be negatively correlated as well as to ensure fairness such that all blocks contribute equally to the solution in opposition to a model dominated by only a few blocks. The tuning parameter, *τ*_*i*_, was selected using the permutation scheme as proposed in Ref.^134^. We have used the RGCCA R permute function to automatically select the hyper-parameters. Bootstrap confidence intervals and p-values were computed for evaluating the significance and stability of the block-weight vectors on 1000 bootstrap samples. The p-value was computed by assuming that the ratio of the blocks weight values to the bootstrap estimate of the standard deviation follows the standardized normal distribution. For a random selection of the variable within the block, the number of occurrences (0 or 1) follows a Bernoulli distribution with the parameter, p = proportion of selected variables in the block. This proportion was estimated by the average number of selected variables over all bootstraps divided by the total number of variables in each block (*pi*). On a larger number of bootstrap samples, the number of occurrences follows a binomial distribution B(n,p) with n = number of bootstraps. The test was based on the following null hypothesis: "the variable is randomly selected according to B(n,p)". This hypothesis was rejected when the number of occurrences is higher than the 1-(0.05/*p*_*i*_)th quantile.

### Limitations of the study

While this study provides important insights into the association of gut microbiota community with estradiol treatment, HFD and anxiety-like behavior, the functional implications of the associations of these microbes identified here remain to be investigated by manipulation studies. The present analyses do not discriminate between effects of HFD-feeding in vehicle-treated animals and the potential independent effects of excess caloric intake in this group. In addition, gut microbes, such as *Akkermansia*, had different associations with anxiety-like behavior in females in the current study compared to those previously reported in males, suggesting a sex difference in their functions and interactions with the host. In future studies, it will be critical to explore these potentially important sex differences in studies using male and female mice.

### Inclusion and diversity

One or more of the authors of this paper self-identifies as an underrepresented ethnic minority in science. One or more of the authors of this paper self-identifies as a member of the LGBTQ + community. One or more of the authors of this paper received support from a program designed to increase minority representation in science.

## Supplementary Information

Supplementary Information.

## Data Availability

No original codes were used in the data analyzed in this paper. All data and any additional information required to reanalyze the data reported in this paper will be shared by the corresponding author upon request.
