# Supplementary material for: Estradiol-mediated protection against high-fat diet induced anxiety and obesity is associated with changes in the gut microbiota in female mice
Source: Sci Rep. 2023 Mar 23;13:4776. doi: 10.1038/s41598-023-31783-6 (PMC10036463; doi:10.1038/s41598-023-31783-6)
Supplement: Supplementary file 1 — Supplementary Information. [file 41598_2023_31783_MOESM1_ESM.docx]

**Supplementary Materials**

**
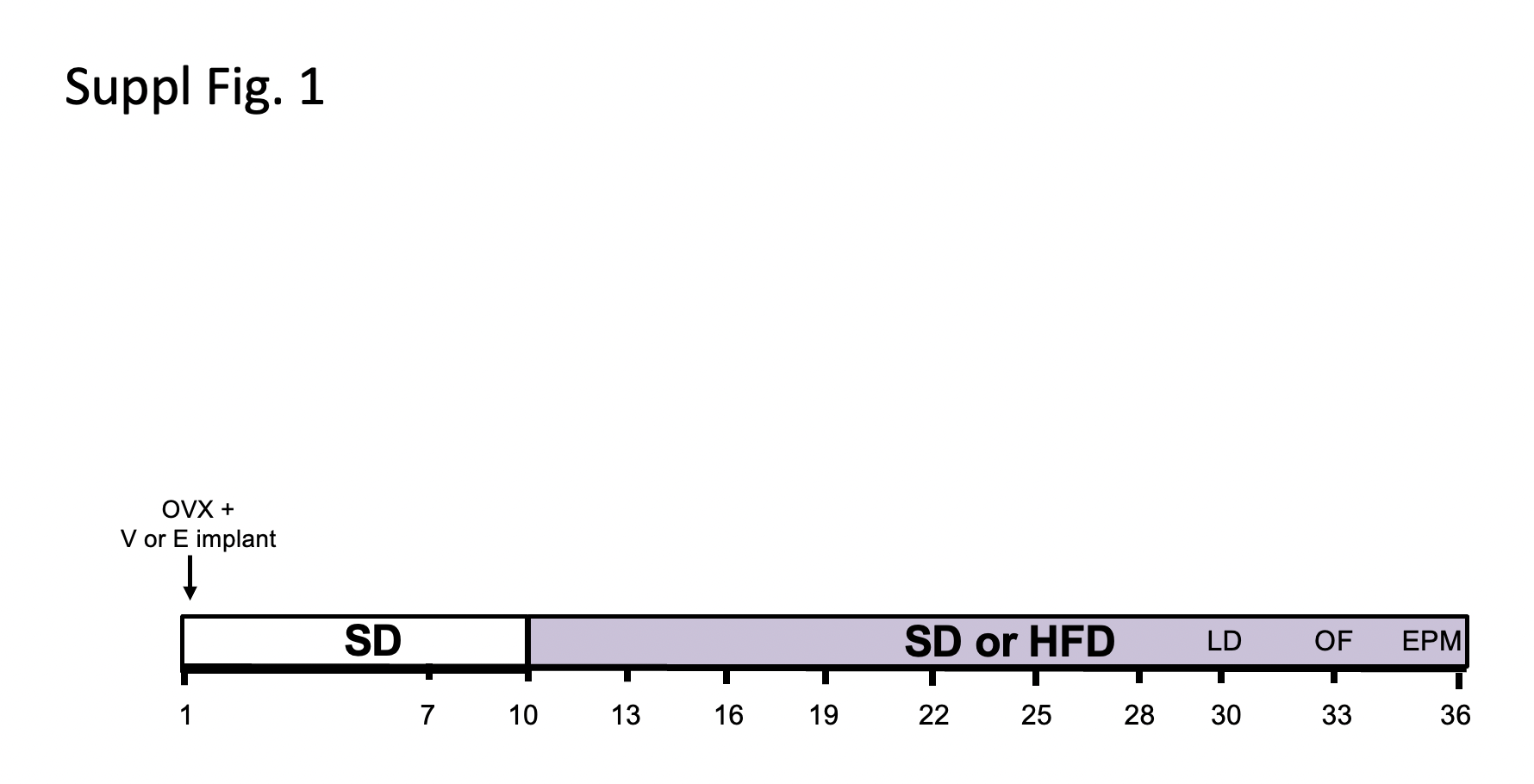
**

**Supplemental Figure 1. Study design.** Eight-week-old female C57BL/6J mice were cohoused, ovariectomized (OVX) and received subcutaneous implants containing vehicle (V) (n=32) or estradiol (E) (n=32). Mice were fed a standard diet (SD) for 10 days and then maintained on SD (n=16) or switched to high fat diet (HFD) (n=16) (pink bar) for 26 more days. Testing for anxiety-like behavior began on day 30 post-OVX with mice tested on the Light-Dark test (LD) on day 30, Open Field test (OF) on day 33, and the Elevated Plus Maze test (EPM) on day 36. Mice were weighed, food intake was measured, and fresh fecal samples were collected every 3 days starting on day 7 post-OVX.


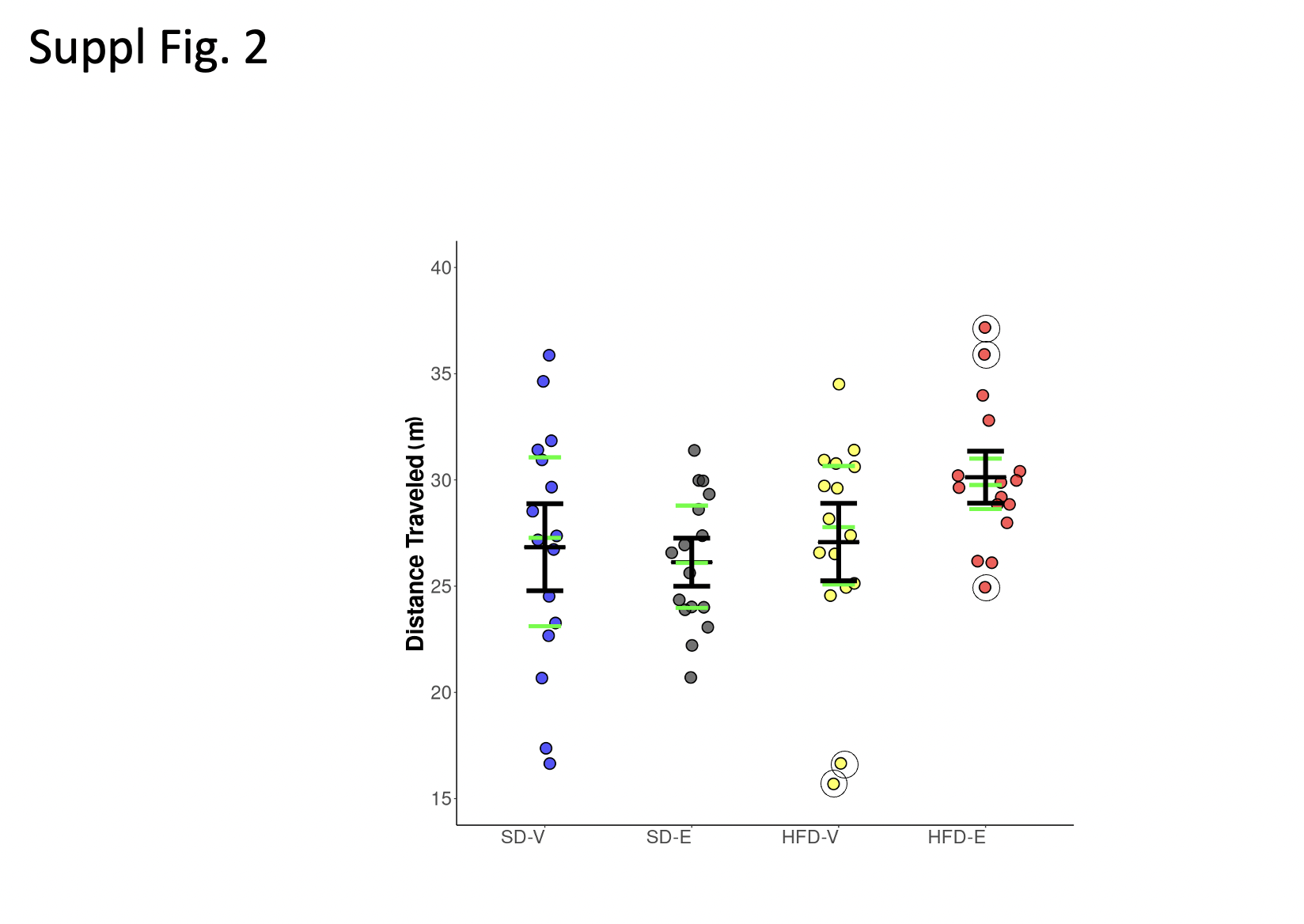


**Supplemental Figure 2. Neither estradiol nor HFD influences locomotor activity on the Open Field test.** The distance traveled during the 10-min testing (n=15-16/group). Black lines show mean (middle line) and 83% CI (whiskers). Green lines show median (middle line) and the 1^st^ (lower line) and the 3^rd^ quartile (upper line). Data points greater than 1.5 times the interquartile range are shown within open circles.


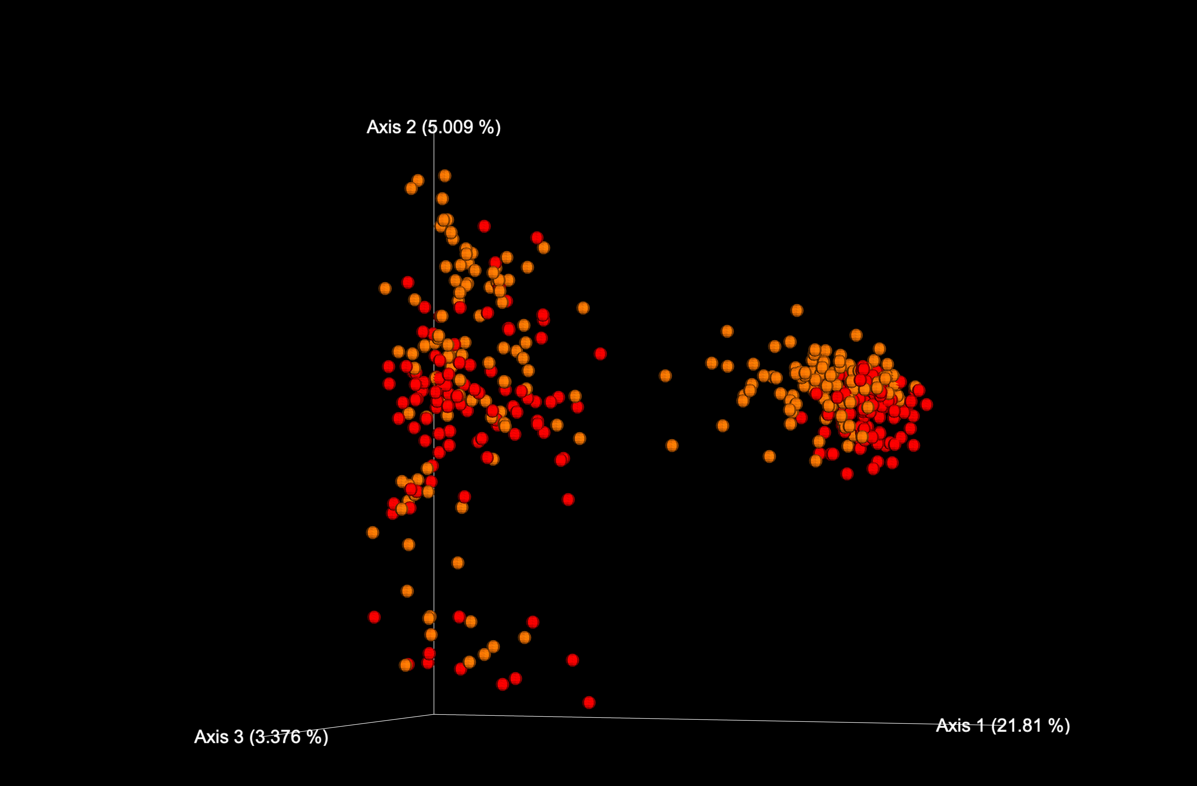


**Supplemental Figure 3. Gut microbial community cluster differently as an effect of estradiol treatment.** Microbial taxa from E (red) or Veh (orange) animals, starting on day 13 (3 days after HFD) were clustered to calculate the Bray-Curtis distance.


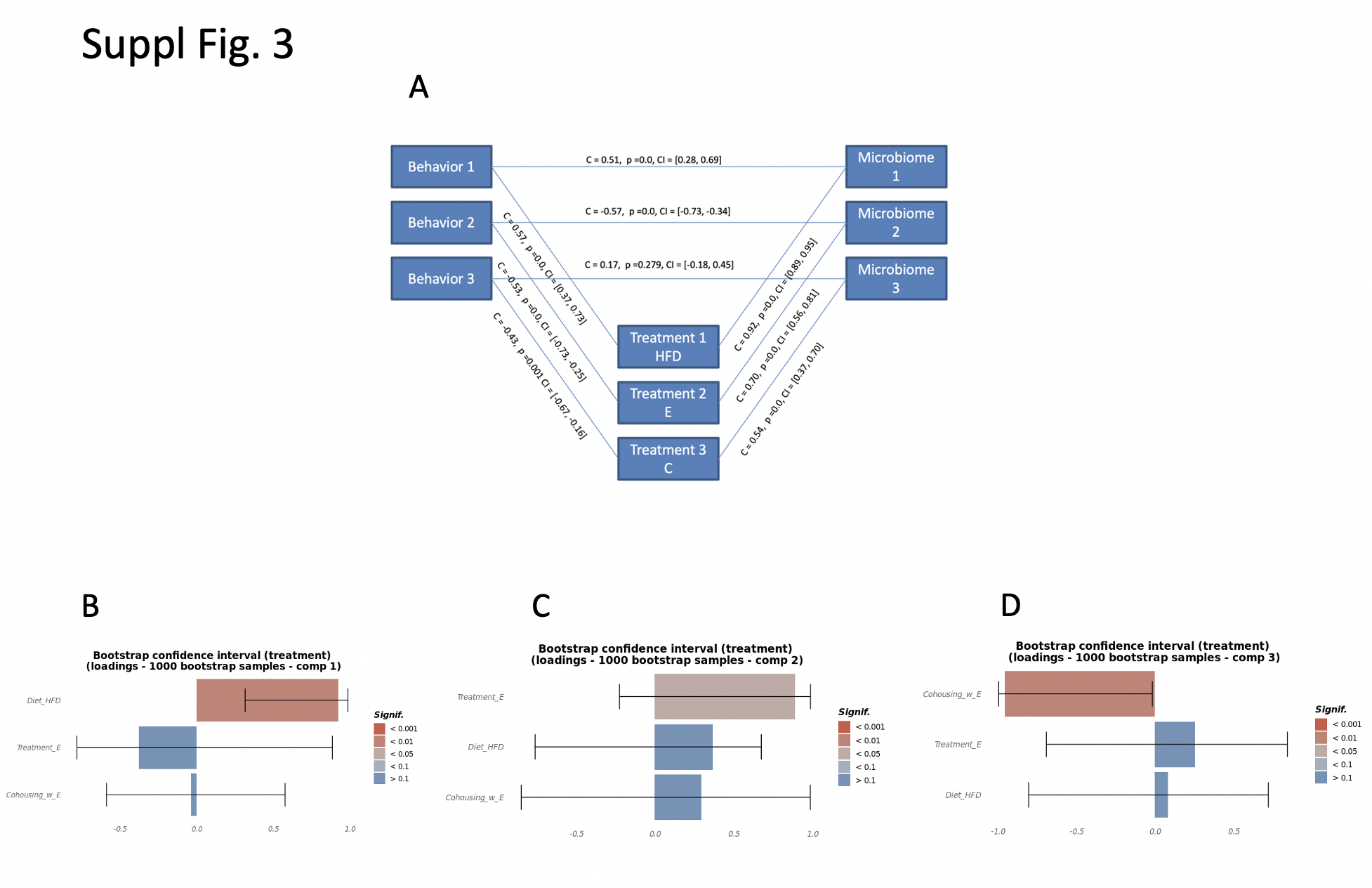


**Supplemental Figure 4. Multiblock generalized canonical correlations between behavioral tests, microbiome abundance features and treatment variables.** (A) Canonical correlations between each block components: microbiome, anxiety-like behavior and treatment variables (HFD; E; C (cohousing with E-treated mice)) are shown. (B) Canonical loadings of treatment variables show that the 1^st^ canonical component is strongly associated with HFD, (C) the 2^nd^ canonical component with E treatment, and (D) the 3^rd^ canonical component with cohousing with E-treated mice (n=8/group). Color gradings depict the statistical significance levels of canonical loadings and whiskers show 95% CI (mean + SEM) which measure the significance and stability of the block-weight vectors on 1000 bootstrap samples. The direction (+/-) of canonical loadings depict the direction (+/-) of variable (anxiety, microbiome, treatment) correlations with canonical variates (anxiety, microbiome, treatment).


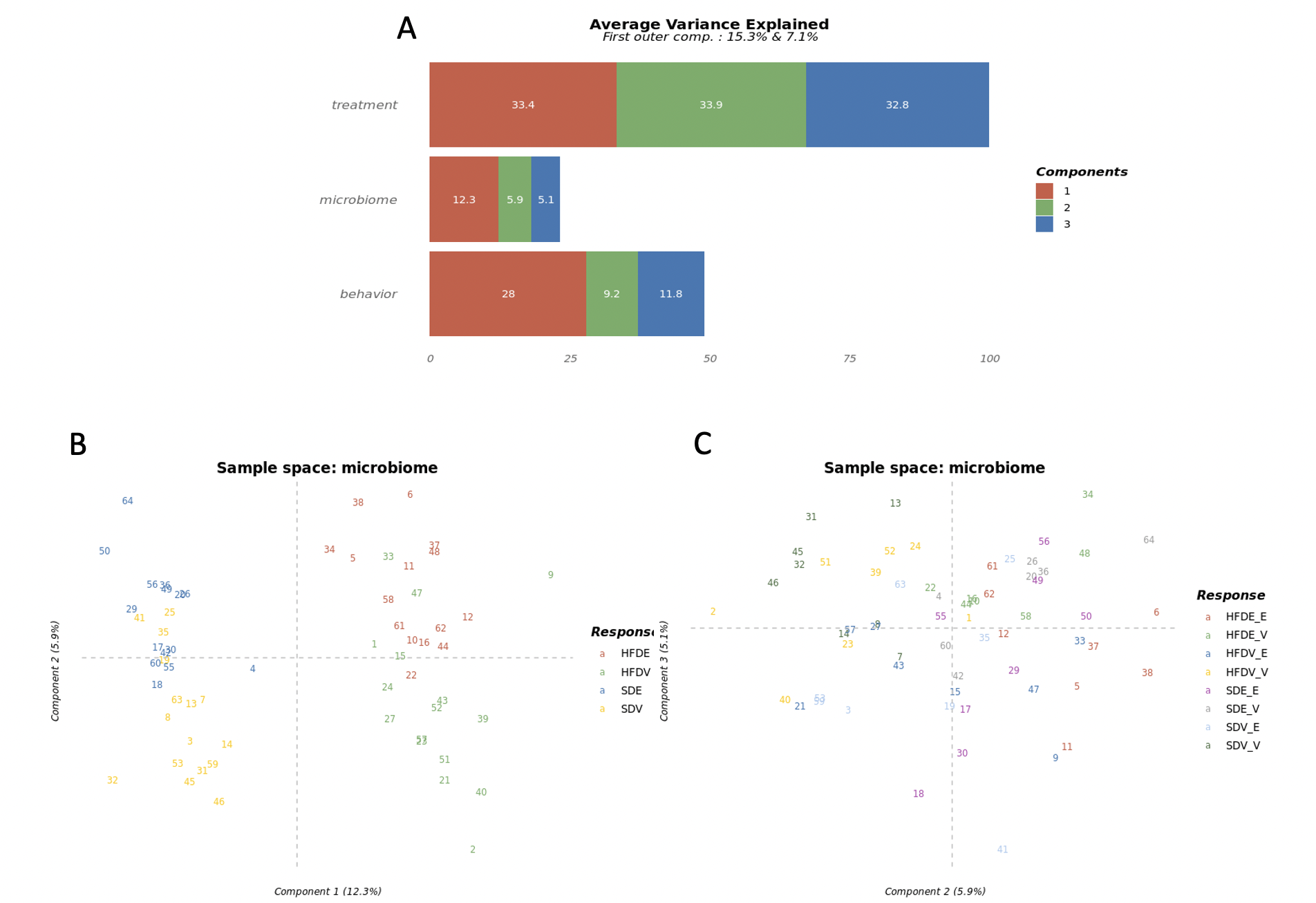


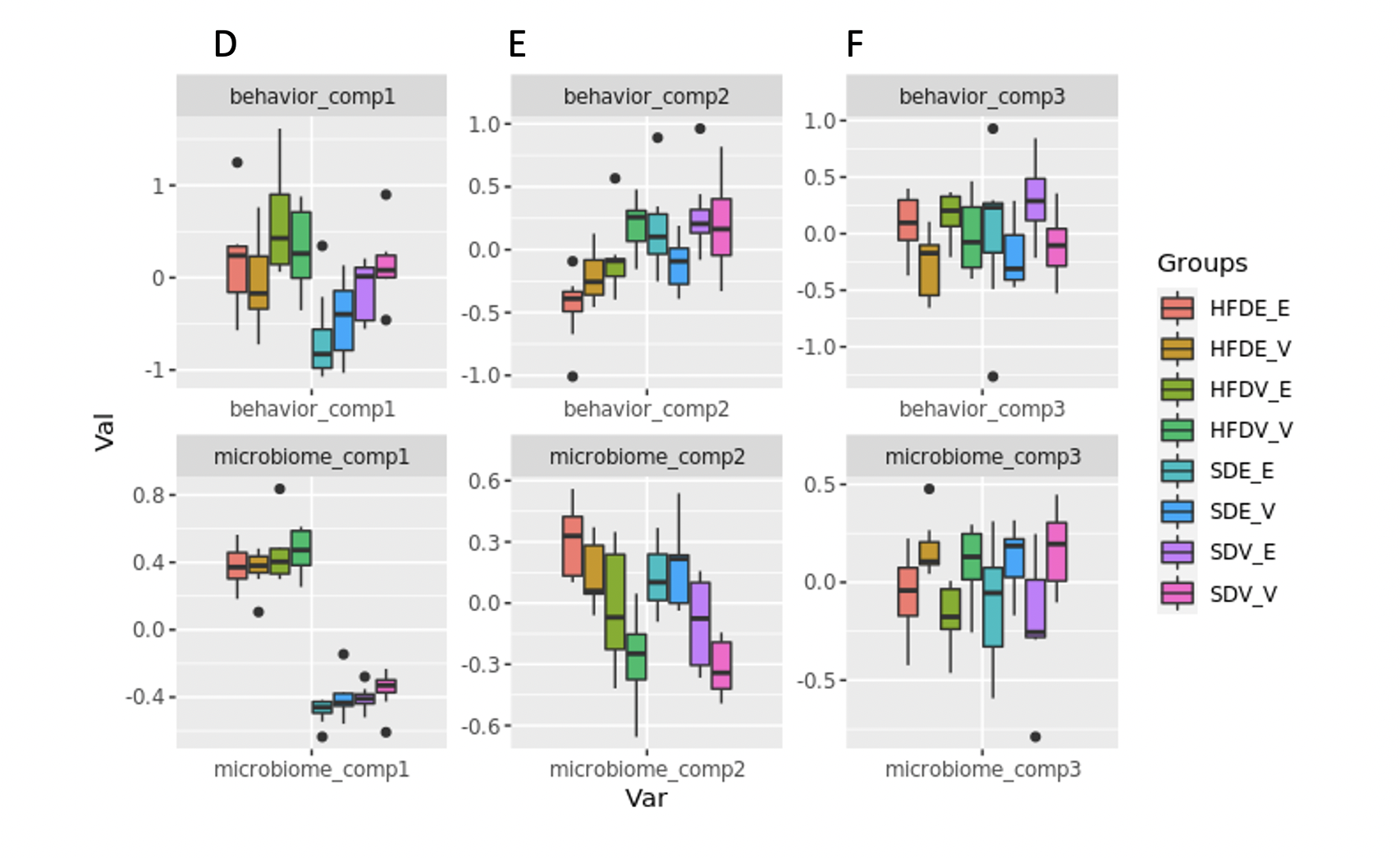


**Supplemental Figure 5. Contribution of the canonical components in explaining variance in behavior, microbiome and treatment blocks.** (A, D) The three components that were derived using individual anxiety measures from the Open Field, Light Dark and Elevated Plus Maze tests explained 28%, 9% and 12% of variance on the anxiety-like behavior. The three components also explained 12%, 6% and 5% of the variance in gut microbiota, respectively. (B, D) The 1^st^ canonical component separated gut microbiota primarily based on diet. (C, E, F) The 2^nd^ and 3^rd^ canonical components were contributed by changes in gut microbiota as a result of E treatment and cohousing with E-treated mice, respectively. HFDE_E: HFD-fed E-treated mice cohoused with partners from the same treatment group; HFDE_V: HFD-fed E-treated mice cohoused with HFD-fed V mice; HFDV_E: HFD-fed V mice cohoused with HFD-fed E-treated mice; HFDV_V: HFD-fed V mice cohoused with partners from the same treatment group; SDE_E: SD-fed E-treated mice cohoused with partners from the same treatment group; SDE_V: SD-fed E-treated mice cohoused with SD-fed V mice; SDV_E: SD-fed V mice cohoused with SD-fed E-treated mice; SDV_V: SD-fed V mice cohoused with partners from the same treatment group.

**
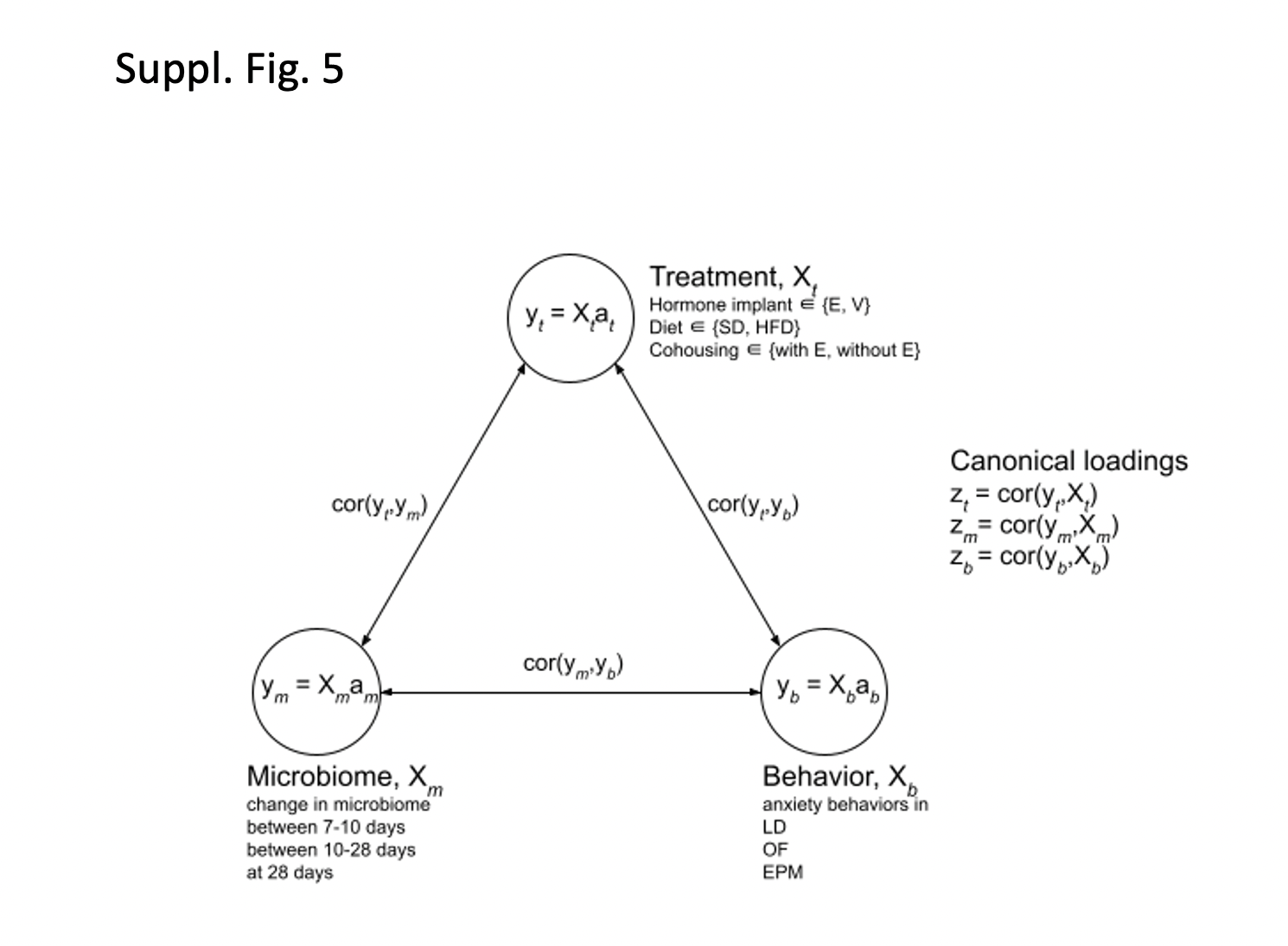
**

**Supplemental Figure 6. RGCCA model description. X*_t_*, X*_m_* and X*_b_* represent the treatment, microbiome and behavior block data matrices**. **a*_t_***, **a*_m_*** and **a*_b_*** represent the weights that transform the data matrices into canonical covariates **y*_t_***, **y*_m_*** and **y*_b_***. The equation maximizes the covariance between each pair of canonical covariates subject to constraints in equation (1). **z_t_**, **z_m_** and **z_b_** represent the canonical loadings. The treatment block has three variables: treatment with E, HFD and cohousing with E mice. The microbiome block has three sets of variables: change in taxa abundance pre-HFD switch (between 7-10 days), post-HFD switch (between 10-28 days) and taxa abundance at 28 days. The behavior block has three sets of variables: behavior tests assessed in LD, OF and EPM tests.

**Supplemental Table 1.** Behavioral measures and their correlations with the 1^st^ canonical component of anxiety.

| Behavior | estimate | mean | sd | lower_bound | upper_bound | bootstrap_ratio | pval | adjust.pval |
| --- | --- | --- | --- | --- | --- | --- | --- | --- |
| LD - Distance travelled in the Light compartment zone (m) | -0.907388 | -0.823836 | 0.354896 | -0.94764674 | -0.3159791 | 4.261912654 | 2.03E-05 | 1.59E-04 |
| LD - Total distance travelled (m) | -0.907388 | -0.823836 | 0.354896 | -0.94764674 | -0.3159791 | 4.261912654 | 2.03E-05 | 1.59E-04 |
| LD - Average speed (m/s) | -0.901306 | -0.818609 | 0.349122 | -0.94427624 | -0.3190632 | 4.236736956 | 2.27E-05 | 1.59E-04 |
| LD - Time in the Light compartment zone (s) | -0.899167 | -0.811576 | 0.360652 | -0.94826007 | -0.289902 | 4.069995315 | 4.70E-05 | 2.47E-04 |
| LD - Average duration of visit to the dark compartment zone (s) | 0.718373 | 0.705001 | 0.299217 | 0.1843306 | 0.8872865 | 3.022139655 | 0.00251 | 0.00824 |
| LD - Longest visit to the dark compartment zone (s) | 0.7007253 | 0.655369 | 0.290083 | 0.12235404 | 0.8531299 | 2.99474504 | 0.002747 | 0.00824 |
| LD - Time oriented towards centre of Light compartment zone when inside zone (s) | -0.673985 | -0.61949 | 0.211507 | -0.79244274 | -0.2162785 | 3.867539142 | 1.10E-04 | 4.62E-04 |
| LD - Number of entries to the Light compartment zone | -0.594935 | -0.573479 | 0.281841 | -0.80265128 | 0.0064913 | 2.431409381 | 0.01504 | 0.039481 |
| EPM - Longest visit to the open arms zone (s) | -0.476007 | -0.410557 | 0.282423 | -0.73820808 | 0.1705312 | 1.833447858 | 0.066736 | 0.155717 |
| EPM - Average duration of visit to the open arms zone (s) | -0.393914 | -0.315635 | 0.267038 | -0.65744913 | 0.2977766 | 1.559416374 | 0.118898 | 0.249686 |
| EPM - Time in the open arms zone (s) | -0.388803 | -0.356009 | 0.293056 | -0.71907586 | 0.2392675 | 1.400379051 | 0.1614 | 0.299102 |
| EPM - Distance travelled in the open arms zone (m) | -0.345375 | -0.312425 | 0.263048 | -0.6772733 | 0.2406304 | 1.369267731 | 0.170916 | 0.299102 |
| EPM - Latency to first entry to the closed arms zone (s) | -0.27903 | -0.255275 | 0.217328 | -0.60739707 | 0.1799006 | 1.31887962 | 0.187209 | 0.302415 |
| EPM - Number of entries to the open arms zone | -0.150817 | -0.186945 | 0.286373 | -0.58973795 | 0.4296253 | 0.530693867 | 0.595631 | 0.827068 |
| EPM - Total distance travelled (m) | -0.120059 | -0.142836 | 0.28267 | -0.59744618 | 0.3796673 | 0.426791818 | 0.669531 | 0.827068 |
| OF - Distance travelled in the center zone (m) | -0.105533 | -0.134175 | 0.243744 | -0.53070788 | 0.3662967 | 0.434584549 | 0.663864 | 0.827068 |
| OF - Time in the center zone (s) | -0.101372 | -0.098843 | 0.226176 | -0.50918853 | 0.3315473 | 0.449743998 | 0.652895 | 0.827068 |
| EPM - Average duration of visit to the closed arms zone (s) | -0.093404 | -0.007695 | 0.29945 | -0.53510064 | 0.5123206 | 0.312829223 | 0.75441 | 0.880145 |
| OF - Total distance travelled (m) | -0.055768 | -0.101181 | 0.309789 | -0.62030681 | 0.4755152 | 0.180206441 | 0.856991 | 0.923794 |
| LD - Average freezing score | 0.0361201 | 0.036654 | 0.238965 | -0.40136523 | 0.4471966 | 0.151217977 | 0.879804 | 0.923794 |
| EPM - Average freezing score | 0.0036429 | 0.019016 | 0.173365 | -0.30739526 | 0.3533125 | 0.021012705 | 0.983236 | 0.983236 |

**Supplemental Table 2.** Gut microbiota taxa and their correlations with 1^st^ canonical component of anxiety.

| **Microbiome** | **estimate** | **mean** | **sd** | **lower_bound** | **upper_bound** | **bootstrap_ratio** | **pval** | **adjust.pval** |
| --- | --- | --- | --- | --- | --- | --- | --- | --- |
| f__Ruminococcaceae_28 | 0.840267985 | 0.817256352 | 0.177298596 | 0.683623525 | 0.909515852 | 6.892804088 | 5.47E-12 | 2.95E-10 |
| g__Lactococcus_28 | 0.836593421 | 0.797286968 | 0.192536639 | 0.634454079 | 0.90599772 | 6.283028269 | 3.32E-10 | 1.03E-08 |
| f__S24_7_28 | -0.811864229 | -0.772293526 | 0.189069338 | -0.878685541 | -0.579123881 | 5.989727619 | 2.10E-09 | 3.78E-08 |
| s__muciniphila_28 | 0.809470212 | 0.797334458 | 0.21569358 | 0.632237416 | 0.910301372 | 5.218006086 | 1.81E-07 | 1.63E-06 |
| f__Erysipelotrichaceae_28 | 0.787759606 | 0.770623793 | 0.170175566 | 0.628623686 | 0.87956734 | 6.261177762 | 3.82E-10 | 1.03E-08 |
| g__Coprococcus_28 | 0.769617498 | 0.745209243 | 0.173235704 | 0.570809263 | 0.865782949 | 5.884403953 | 3.99E-09 | 5.39E-08 |
| g__Anaeroplasma_28 | -0.73185762 | -0.699770397 | 0.153840735 | -0.824550774 | -0.529483212 | 6.062866807 | 1.34E-09 | 2.89E-08 |
| f__Mogibacteriaceae_28 | 0.725295833 | 0.727600943 | 0.133020853 | 0.602529539 | 0.832160382 | 6.906657926 | 4.96E-12 | 2.95E-10 |
| g__rc4_4_28 | 0.718375742 | 0.684441452 | 0.188212057 | 0.446083263 | 0.842881233 | 4.804583339 | 1.55E-06 | 1.29E-05 |
| g__Coprobacillus_28 | 0.688646115 | 0.666609614 | 0.155875908 | 0.490015008 | 0.809091285 | 5.423391723 | 5.85E-08 | 5.74E-07 |
| f__Peptostreptococcaceae_28 | 0.663500523 | 0.618551175 | 0.203430174 | 0.34183648 | 0.814953491 | 3.927841148 | 8.57E-05 | 5.79E-04 |
| g__Turicibacter_28 | -0.663227648 | -0.640676587 | 0.134636566 | -0.775946279 | -0.476209856 | 5.931182791 | 3.01E-09 | 4.64E-08 |
| g__Lachnospira_28 | -0.636887333 | -0.623332935 | 0.130883743 | -0.75908659 | -0.457228398 | 5.752580674 | 8.79E-09 | 1.05E-07 |
| s__piliforme_28 | -0.612623619 | -0.605040026 | 0.129921834 | -0.748549455 | -0.445690565 | 5.488765366 | 4.05E-08 | 4.37E-07 |
| g__Adlercreutzia_28 | 0.602487233 | 0.578443372 | 0.159248303 | 0.356054154 | 0.746854674 | 4.377080152 | 1.20E-05 | 8.66E-05 |
| o__RF39_28 | -0.589493405 | -0.592660107 | 0.143080705 | -0.760470414 | -0.426573665 | 4.730821793 | 2.24E-06 | 1.73E-05 |
| g__Ruminococcus_28 | -0.543335441 | -0.551257781 | 0.180730342 | -0.759076322 | -0.312101922 | 3.368975281 | 7.54E-04 | 0.004074203 |
| g__Oscillospira_28 | 0.533593151 | 0.488488254 | 0.214366795 | 0.160596881 | 0.753665969 | 2.776340448 | 0.005497461 | 0.024738575 |
| f__Erysipelotrichaceae_28 _13_change | 0.52444952 | 0.514251903 | 0.155774701 | 0.286234162 | 0.711865545 | 3.739103999 | 1.85E-04 | 0.001049745 |
| g__Adlercreutzia_28 _13_change | 0.518623598 | 0.509531187 | 0.148903657 | 0.273768759 | 0.688375015 | 3.857897761 | 1.14E-04 | 6.86E-04 |
| g__Lactobacillus_28 | -0.506642726 | -0.482442375 | 0.143802967 | -0.678451957 | -0.253264333 | 3.881719242 | 1.04E-04 | 6.59E-04 |
| g__Ruminococcus_28 _13_change | -0.500105125 | -0.492695879 | 0.189805605 | -0.732942863 | -0.194161009 | 2.894784479 | 0.003794189 | 0.017816194 |
| f__Lachnospiraceae_28 | -0.432264383 | -0.43516732 | 0.192082894 | -0.696476202 | -0.116989012 | 2.408741586 | 0.016007628 | 0.061743707 |
| s__gnavus_28 | 0.424642251 | 0.396044652 | 0.16582149 | 0.086173718 | 0.632500968 | 2.733915823 | 0.006258604 | 0.02703717 |
| g__Dorea_28 | 0.38886071 | 0.359343814 | 0.158671455 | 0.05748144 | 0.598649884 | 2.586836273 | 0.00968616 | 0.040234821 |
| g__Roseburia_28 | -0.381078477 | -0.37601194 | 0.124326064 | -0.588182795 | -0.180699092 | 3.227969544 | 0.001246722 | 0.006411716 |
| f__Lachnospiraceae_28 _13_change | -0.378101116 | -0.357371337 | 0.170016015 | -0.601948948 | -0.053571865 | 2.340027534 | 0.019282318 | 0.069416346 |
| s__muciniphila_28 _13_change | 0.375662631 | 0.356095759 | 0.20581956 | -0.024727324 | 0.659296943 | 1.919156589 | 0.05496452 | 0.169604803 |
| f__Christensenellaceae_28 _13_change | 0.374873795 | 0.368283067 | 0.178433383 | 0.058181616 | 0.63018391 | 2.2085656 | 0.02720487 | 0.094778258 |
| g__Anaerotruncus_28 | 0.363858265 | 0.371377744 | 0.124211348 | 0.175577208 | 0.57707102 | 3.069975262 | 0.002140765 | 0.01050921 |
| s__piliforme_10 _07_change | 0.350101267 | 0.314592128 | 0.153497527 | 0.014532307 | 0.548321445 | 2.381531279 | 0.017240827 | 0.064207216 |
| g__Oscillospira_28 _13_change | -0.334061952 | -0.324642244 | 0.204588821 | -0.639192046 | 0.030417885 | 1.69800827 | 0.089506201 | 0.252514023 |
| g__Dehalobacterium_28 _13_change | -0.323983696 | -0.310593088 | 0.198967735 | -0.629628436 | 0.029913954 | 1.689176457 | 0.091185619 | 0.252514023 |
| g__Roseburia_28 _13_change | -0.307583488 | -0.308050075 | 0.128019321 | -0.540719714 | -0.110761528 | 2.483017304 | 0.013027476 | 0.052109904 |
| g__Turicibacter_10 _07_change | 0.29130449 | 0.267854334 | 0.144953936 | 1.46E-04 | 0.510808821 | 2.069561763 | 0.038493402 | 0.129915232 |
| s__ovatus_10 _07_change | -0.289894032 | -0.218789175 | 0.211571764 | -0.555381704 | 0.196058445 | 1.410635159 | 0.158352219 | 0.363765926 |
| g__Turicibacter_28 _13_change | -0.277780182 | -0.261259883 | 0.176084651 | -0.554899362 | 0.044393496 | 1.620101661 | 0.105210441 | 0.28406819 |
| g__Anaeroplasma_28 _13_change | -0.272882435 | -0.250821584 | 0.185652128 | -0.547229928 | 0.087219667 | 1.508065302 | 0.131537815 | 0.338240095 |
| f__Peptostreptococcaceae_28 _13_change | 0.260850858 | 0.257480784 | 0.134214117 | -0.012313283 | 0.489067686 | 1.989516267 | 0.046644248 | 0.150111738 |
| g__Lachnospira_28 _13_change | 0.255069772 | 0.243985339 | 0.137351687 | -0.036704955 | 0.477347523 | 1.898978795 | 0.057567264 | 0.172701792 |
| f__S24_7_28 _13_change | 0.252444443 | 0.236038447 | 0.166304148 | -0.074855717 | 0.515322712 | 1.551506247 | 0.120780411 | 0.318153279 |
| g__Sutterella_28 | -0.248235578 | -0.194479325 | 0.208024039 | -0.536688757 | 0.206719667 | 1.218761286 | 0.222934809 | 0.445869618 |
| f__Ruminococcaceae_28 _13_change | -0.248029303 | -0.266533529 | 0.178474841 | -0.563086934 | 0.046323274 | 1.419313978 | 0.155807499 | 0.363765926 |
| o__Streptophyta_28 | -0.232524374 | -0.171621627 | 0.119384148 | -0.404343156 | 0 | 1.983986015 | 0.047257399 | 0.150111738 |
| g__Lactobacillus_28 _13_change | -0.217795794 | -0.19095583 | 0.158161192 | -0.456326343 | 0.114799158 | 1.399464473 | 0.161673745 | 0.363765926 |
| f__Ruminococcaceae_10 _07_change | 0.217256804 | 0.177516442 | 0.161430683 | -0.132804711 | 0.449035904 | 1.367616151 | 0.171432258 | 0.377850691 |
| g__Anaerotruncus_28 _13_change | -0.215482027 | -0.202515221 | 0.155987785 | -0.475770769 | 0.104792785 | 1.403400028 | 0.160497585 | 0.363765926 |
| f__Clostridiaceae_28 | -0.213322099 | -0.204009075 | 0.146382271 | -0.471277723 | 0.06842609 | 1.480023906 | 0.138866867 | 0.348781898 |
| f__Mogibacteriaceae_10 _07_change | 0.210623537 | 0.190525763 | 0.147509432 | -0.089516685 | 0.440000768 | 1.449559777 | 0.147181319 | 0.361263239 |
| s__piliforme_28 _13_change | -0.210211547 | -0.21367204 | 0.163549046 | -0.505693059 | 0.072123252 | 1.304762511 | 0.191973728 | 0.40132887 |
| g__Anaerostipes_28 _13_change | 0.204226526 | 0.201248875 | 0.159205983 | -0.102355565 | 0.478897239 | 1.301076066 | 0.193232419 | 0.40132887 |
| o__Clostridiales_28 | -0.203138878 | -0.220190452 | 0.208475396 | -0.571965707 | 0.164576058 | 0.988147157 | 0.323080584 | 0.601598328 |
| g__Anaeroplasma_10 _07_change | -0.195424957 | -0.183804822 | 0.149339344 | -0.438471763 | 0.111280503 | 1.325647891 | 0.184956355 | 0.399505728 |
| g__Coprococcus_28 _13_change | 0.185475471 | 0.187616937 | 0.14658212 | -0.07844419 | 0.438537456 | 1.280151605 | 0.200491823 | 0.408549374 |
| g__Lactococcus_10 _07_change | 0.181143511 | 0.107653596 | 0.107545402 | 0 | 0.352034075 | 1.703138756 | 0.088542105 | 0.252514023 |
| s__ovatus_28 _13_change | 0.175131816 | 0.148423221 | 0.148940201 | -0.134199213 | 0.424712079 | 1.188101008 | 0.234793617 | 0.461049284 |
| g__Eubacterium_28 _13_change | -0.167721131 | -0.151036264 | 0.155162946 | -0.426676353 | 0.128340757 | 1.091245754 | 0.27516476 | 0.530674895 |
| f__Erysipelotrichaceae_10 _07_change | 0.167070564 | 0.146590445 | 0.174887685 | -0.240646123 | 0.424465602 | 0.964342258 | 0.334874361 | 0.60277385 |
| o__RF39_10 _07_change | -0.16274487 | -0.156557336 | 0.157162208 | -0.430044741 | 0.141400929 | 1.04481199 | 0.296109872 | 0.561050285 |
| s__gnavus_28 _13_change | -0.15499686 | -0.13401989 | 0.189122189 | -0.454946499 | 0.233071568 | 0.826218652 | 0.40868007 | 0.696281471 |
| g__Oscillospira_10 _07_change | 0.151720432 | 0.092920853 | 0.240167784 | -0.38141658 | 0.498323785 | 0.636642153 | 0.524357961 | 0.768671451 |
| f__Lachnospiraceae_10 _07_change | 0.145666982 | 0.080296965 | 0.231741507 | -0.365996366 | 0.495030666 | 0.633078639 | 0.526682291 | 0.768671451 |
| o__Clostridiales_10 _07_change | 0.143009379 | 0.091694681 | 0.203584168 | -0.294768518 | 0.457556305 | 0.707306706 | 0.479375899 | 0.761643136 |
| f__Peptostreptococcaceae_10 _07_change | 0.140252124 | 0.091981843 | 0.146144077 | -0.207225166 | 0.354426776 | 0.966051804 | 0.33401826 | 0.60277385 |
| f__S24_7_10 _07_change | -0.138579629 | -0.083247971 | 0.214850639 | -0.462234827 | 0.312746302 | 0.649181696 | 0.516220941 | 0.768671451 |
| g__Ruminococcus_10 _07_change | 0.135395137 | 0.116266796 | 0.168591892 | -0.214712756 | 0.422674489 | 0.808056092 | 0.419058293 | 0.696281471 |
| g__Anaerostipes_28 | -0.134739681 | -0.105584671 | 0.15951933 | -0.3769102 | 0.225941087 | 0.849828479 | 0.395420454 | 0.696281471 |
| g__Coprobacillus_28 _13_change | -0.134500804 | -0.14221053 | 0.212327542 | -0.51242927 | 0.281090023 | 0.637320919 | 0.523915828 | 0.768671451 |
| g__cc_115_28 | 0.130384134 | 0.120047403 | 0.194933251 | -0.243216634 | 0.465966208 | 0.672694915 | 0.501141404 | 0.768671451 |
| o__Streptophyta_10 _07_change | 0.127886592 | 0.093453217 | 0.157219716 | -0.218312235 | 0.367182272 | 0.817904471 | 0.413411737 | 0.696281471 |
| o__Streptophyta_28 _13_change | 0.122759715 | 0.123890124 | 0.152181756 | -0.190001819 | 0.390543062 | 0.810754295 | 0.417506787 | 0.696281471 |
| s__gnavus_10 _07_change | 0.114768651 | 0.067458091 | 0.201010808 | -0.308562385 | 0.444714037 | 0.573484468 | 0.566316713 | 0.804765855 |
| g__Clostridium_10 _07_change | -0.109836233 | -0.106722386 | 0.139364509 | -0.374232073 | 0.157001116 | 0.791314423 | 0.428760534 | 0.701608146 |
| g__Dehalobacterium_10 _07_change | 0.109755577 | 0.056250796 | 0.220477025 | -0.369645307 | 0.446981686 | 0.499823082 | 0.617199656 | 0.812897108 |
| f__Clostridiaceae_10 _07_change | -0.104817523 | -0.110427803 | 0.148798774 | -0.389186748 | 0.164593178 | 0.70702155 | 0.479553086 | 0.761643136 |
| f__Clostridiaceae_28 _13_change | -0.100259663 | -0.097875414 | 0.154026509 | -0.373244697 | 0.193863414 | 0.653119007 | 0.513679559 | 0.768671451 |
| g__Bacteroides_28 _13_change | 0.09873092 | 0.098657948 | 0.168035462 | -0.22988956 | 0.395768886 | 0.589480405 | 0.555539053 | 0.799976236 |
| g__Anaerotruncus_10 _07_change | 0.096063417 | 0.060960639 | 0.172261127 | -0.283685165 | 0.391886703 | 0.559386567 | 0.575897927 | 0.807752937 |
| s__muciniphila_10 _07_change | -0.091999178 | -0.077304674 | 0.17488694 | -0.410133559 | 0.235808003 | 0.527541165 | 0.597817837 | 0.812897108 |
| f__Mogibacteriaceae_28 _13_change | -0.089946892 | -0.040949365 | 0.202718459 | -0.402342012 | 0.321244081 | 0.444905941 | 0.656387729 | 0.833998526 |
| g__Lactobacillus_10 _07_change | -0.087897846 | -0.050542721 | 0.19322146 | -0.405262821 | 0.322470718 | 0.456084266 | 0.648329384 | 0.833998526 |
| g__Eubacterium_28 | -0.087734057 | -0.061266604 | 0.170622812 | -0.3660945 | 0.244522264 | 0.515524307 | 0.606186689 | 0.812897108 |
| g__Clostridium_28 _13_change | 0.084153464 | 0.032686031 | 0.16874119 | -0.336442873 | 0.332839735 | 0.499895525 | 0.617148643 | 0.812897108 |
| g__Sutterella_10 _07_change | 0.082217779 | 0.116692958 | 0.19117783 | -0.234455754 | 0.485588839 | 0.431032151 | 0.666444993 | 0.836930922 |
| g__Eubacterium_10 _07_change | 0.07861115 | 0.058093564 | 0.151859513 | -0.237291109 | 0.355516623 | 0.51872736 | 0.603950879 | 0.812897108 |
| g__Sutterella_28 _13_change | -0.074558822 | -0.039818391 | 0.182703699 | -0.358652447 | 0.31971855 | 0.408844719 | 0.68265362 | 0.841679146 |
| o__RF39_28 _13_change | 0.073590007 | 0.081282366 | 0.163214405 | -0.2262008 | 0.406265974 | 0.451695927 | 0.651488053 | 0.833998526 |
| o__Clostridiales_28 _13_change | 0.069495908 | 0.080252402 | 0.172065567 | -0.225997047 | 0.404438993 | 0.404544124 | 0.685812638 | 0.841679146 |
| g__Coprococcus_10 _07_change | 0.069335231 | 0.028137394 | 0.200137369 | -0.342921512 | 0.387500068 | 0.346994963 | 0.728595103 | 0.864706276 |
| g__cc_115_10 _07_change | -0.060977473 | -0.025181541 | 0.16441514 | -0.292609724 | 0.341681173 | 0.371335745 | 0.710387478 | 0.852464973 |
| g__Clostridium_28 | -0.059843494 | -0.055974803 | 0.160834934 | -0.362996876 | 0.246956979 | 0.372525324 | 0.709501761 | 0.852464973 |
| f__Christensenellaceae_28 | -0.055286464 | -0.044997426 | 0.167197201 | -0.353363032 | 0.270710834 | 0.331003726 | 0.740641671 | 0.869448918 |
| g__Roseburia_10 _07_change | 0.051490603 | 0.036597983 | 0.164420464 | -0.290920802 | 0.320868527 | 0.313441401 | 0.753945334 | 0.87554942 |
| g__Dorea_10 _07_change | 0.049140668 | 0.07493255 | 0.170072045 | -0.235024402 | 0.380723721 | 0.289173218 | 0.772448826 | 0.887494396 |
| s__ovatus_28 | -0.045715459 | -0.024226895 | 0.184101504 | -0.34160893 | 0.3403854 | 0.2484898 | 0.803755461 | 0.906871843 |
| g__Adlercreutzia_10 _07_change | 0.045124701 | 0.065724339 | 0.184474155 | -0.25936672 | 0.424890975 | 0.244778826 | 0.806627689 | 0.906871843 |
| g__rc4_4_28 _13_change | 0.038306787 | 0.02386466 | 0.163352968 | -0.313725018 | 0.330004706 | 0.234617963 | 0.814505266 | 0.906871843 |
| g__rc4_4_10 _07_change | 0.036893373 | 0.049385561 | 0.183049678 | -0.282660193 | 0.409231665 | 0.201639936 | 0.840198222 | 0.925932734 |
| g__cc_115_28 _13_change | -0.02448314 | -0.021665953 | 0.181183411 | -0.375749748 | 0.319984777 | 0.135156049 | 0.892488496 | 0.963887576 |
| g__Bacteroides_28 | 0.024094465 | 0.047944981 | 0.147657646 | -0.241069154 | 0.326554637 | 0.16320949 | 0.870353494 | 0.949476539 |
| g__Coprobacillus_10 _07_change | 0.017608968 | 0.042521794 | 0.186288827 | -0.283926377 | 0.41532482 | 0.094534862 | 0.924684291 | 0.979077484 |
| g__Lactococcus_28 _13_change | 0.015940032 | 8.30E-04 | 0.164145249 | -0.300265167 | 0.307330061 | 0.097117538 | 0.922633053 | 0.979077484 |
| f__Christensenellaceae_10 _07_change | 0.008116645 | 0.038413823 | 0.179667177 | -0.300761431 | 0.381472972 | 0.045176994 | 0.963966232 | 0.986439212 |
| g__Dehalobacterium_28 | -0.007331869 | -0.027382594 | 0.182489051 | -0.374576386 | 0.30171075 | 0.040177757 | 0.96795141 | 0.986439212 |
| g__Dorea_28 _13_change | -0.007122637 | -0.017378991 | 0.183966207 | -0.364178201 | 0.306789225 | 0.038717749 | 0.969115422 | 0.986439212 |
| g__Anaerostipes_10 _07_change | 0.005165526 | -0.003896713 | 0.181584825 | -0.326498351 | 0.358414455 | 0.028447154 | 0.977305516 | 0.986439212 |
| g__Bacteroides_10 _07_change | 0.004449666 | -0.036918668 | 0.138985654 | -0.315813835 | 0.202094962 | 0.032015498 | 0.974459692 | 0.986439212 |
| g__Lachnospira_10 _07_change | -0.001446652 | 0.011430056 | 0.157080557 | -0.31104337 | 0.298565879 | 0.009209626 | 0.992651885 | 0.992651885 |

**Supplemental Table 3. High Fat Diet (HFD) and Standard Diet (SD) ingredients and content.**

|  | **Research Diets D12492**  **60% kcal fat High-Fat Diet (HFD)** | **Lab Diet 5001**  **Rodent Laboratory Chow (SD)** |
| --- | --- | --- |
| Main Ingredients | Lard, 30 mesh lactic casein, lodex 10 (proprietary maltodextrin), sucrose, Solka Floc FCC200 (proprietary cellulose), soybean oil, | Ground corn, dehulled soybean meal, dried beet pulp, fish meal, ground oats, brewers dried yeast, cane molasses, dehydrated alfalfa meal, dried whey, wheat germ, porcine animal fat preserved with BHA, porcine meat meal, wheat middlings, salt, |
| Supplements | L-cystine, choline bitartrate, Potassium Citrate Monohydrate, Calcium Phosphate Dibasic, Calcium Carbonate Light USP, Sodium Chloride, Magnesium Sulfate, Heptahydrate, Magnesium Oxide Heavy DC USP, Ferric Citrate, Carbonate Hydrate, Zinc Carbonate, Chromium Potassium Sulfate, Copper Carbonate, Ammonium Molybdate Tetrahydrate, Fluoride, Selenite, Potassium Iodate, Vitamin E Acetate, Niacin, Pantothenic Acid d Calcium, Vitamin D3, Vitamin B12 w/ 0.1% Mannitol, Vitamin A Acetate, Pyridoxine HCl, Riboflavin Thiamine HCl, Folic Acid, Menadione Sodium Bisulfite, 6.5% cellulose by weight as the fiber source | Calcium carbonate, DL-methionine, choline chloride, vitamin D3 supplement, folic acid, vitamin A acetate, menadione dimethylpyrimidinol bisulfite (source of vitamin K), pyridoxine hydrochloride, biotin, thiamine mononitrate, nicotinic acid, calcium pantothenate, vitamin E supplement, vitamin B-12 supplement, riboflavin supplement, ferrous sulfate, manganous oxide, zinc oxide, ferrous carbonate, copper sulfate, zinc sulfate, calcium iodate, cobalt carbonate, sodium selenite, 5.1% crude fiber (cellulose, hemi-cellulose, and lignin) by weight |
| Isoflavones | Commercially produced soybean oil does not contain detectable amounts of isoflavones ^149^. | The soy ingredients contribute 228 ± 52 daidzein, genistein 257 ± 61, 4.7 diethylstilbestrol equivalents (μg/g diet). These levels vary across batches due to seasonal availability of ingredients ^150^ |
